# Robotic surgery across surgical specialties: an Italian survey on implementation, training, and satisfaction levels among trainees and young attendings: on behalf of the Italian Multispecialty Society of Young Surgeons (SPIGC)

**DOI:** 10.1007/s13304-026-02747-y

**Published:** 2026-09-07

**Authors:** Francesco Del Giudice, Valerio Santarelli, Giulia Fiori, Sara Mastrovito, Beatrice Trabalza Marinucci, Sara Mantovani, Alberto Bolletta, Giuseppe Rovere, Danilo Di Giorgio, Fabio Massimo Oddi, Dalila Carino, Mattia Di Bartolomeo, Benjamin I. Chung, Felice Crocetto, Michele Maruccia, Roberto Meniconi, Alessandro Pasculli, Marco Angrisani

**Affiliations:** 1 Department of Maternal Infant and Urologic Sciences, Policlinico Umberto I Hospital, “Sapienza” University of Rome, Rome, Italy; 2 Department of Urology, Stanford University School of Medicine, Stanford, CA USA; 3 The Urology Centre, Guy’s and St Thomas’​ NHS Foundation Trust, London, UK; 4 Department of Surgery, San Giovanni-Addolorata Hospital, Rome, Italy; 5 Department of Obstetrics and Gynecology, Istituto di Ricovero e Cura a Carattere Scientifico, Fondazione Policlinico Universitario Agostino Gemelli, Rome, Italy; 6 Thoracic Surgery, Sant’Andrea Hospital, Sapienza University, Rome, Italy; 7 Department of Thoracic Surgery, Azienda Ospedaliera San Camillo-Forlanini, Rome, Italy; 8 Plastic Surgery and Microsurgery Unit, Department of Translational Research and New Technologies in Medicine and Surgery, University of Pisa, Pisa, Italy; 9 Department of Clinical Science and Translational Medicine, Section of Orthopaedics and Traumatology, University of Rome “Tor Vergata”, Rome, Italy; 10 Department of Oncological and Reconstructive Maxillofacial Surgery, Policlinico Umberto I Hospital, “Sapienza” University of Rome, Rome, Italy; 11 Vascular Surgery Unit, Biomedicine and Prevention Department, “Tor Vergata” University of Rome, Rome, Italy; 12 Unit of Dentistry and Maxillo-Facial Surgery, Surgery, Dentistry, Maternity and Infant Department, University of Verona, P.le L.A. Scuro 10, 37134 Verona, Italy; 13 Department of Neurosciences, Reproductive Sciences and Odontostomatology, University of Naples Federico II, Naples, Italy; 14 Section of Plastic and Reconstructive Surgery, Department of Emergency and Organ Transplantation, University of Bari, Bari, Italy; 15 Department of General Surgery and Transplantation Unit, San Camillo Forlanini Hospital, Rome, Italy; 16 Unit of Academic General Surgery “F.Paccione”, Department of Precision and Regenerative Medicine and Ionian Area (DiMePre-J), University of Bari “A. Moro”, 70125 Bari, Italy

**Keywords:** Robotic surgery, Minimally-invasive surgery, Multispeciality surgical training, Survery SPIGC

## Abstract

European surgical institutions have been recently criticized for the insufficient endorsement of standardized robotic training curricula. In the present study, we report the results of a nationwide survey on the status of robotic surgery training and satisfaction across trainees from multiple surgical specialties in Italy. A modified-Delphi consensus was implemented with *n* = 41 multiple-choice or open-ended question surveys later distributed to Residents, Fellows, PhD students, Young Attendings or Assistant Professors, in *n* = 10 different surgical specialties. Demographics, program characteristics, exposure to robotic surgery, and satisfaction with robotic training were explored. Composite variables were created, and multivariable regression modeling adopted to explore variables associated with higher surgical training satisfaction. Similarly, predictors of minimum requirements necessary for designation as a ‘reference center’ for robotic surgical training institutions were further explored. A total of *n* = 735 trainees participated and were included for final analysis. The vast majority were male (63.5%), residents (52.1%), from University Hospitals (53.7%), and from the Central Regions of Italy (47.9%). General Surgery was the most represented specialty (44.4%) followed by Urology (26%). Availability of a dual robotic console, simulation software, and scheduled lectures were reported in 45.6%, 58.2%, and 23.4% respectively. Most respondents (61.6%) reported that the robotic console was exclusively operated by senior/experienced attendings, and the majority (88.3%) believed that the lack of standardized robotic exposure negatively impacted their training. On univariable analysis, Urology achieved the highest satisfaction with robotic training (median score 6 [IQR: 5–8], *p* < 0.001). However, on multivariable modeling, the surgical specialty did not impact overall satisfaction, which was instead influenced by simulation software availability (adjusted Odds Ratio [aOR]: 1.72, 95% CI 1.22–2.44), dedicated lectures (aOR: 1.72, 95% CI 1.16–2.56), higher volume of robotic-assisted procedures per center (aOR: 9.69, 95% CI 5.62–16.70), and trainee with more active role during robotic cases (aOR: 2.43, 95% CI 1.29–4.57). Finally, centers with urology specialty (*p* < 0.001), > 400 procedures/year (aOR: 1.92, 95% CI 1.01–4.51), > 1 robotic platform (aOR: 1.29, 95% CI 1.07–1.55), and providing robotics fellowship/masters programs (aOR: 1.43, 95% CI 0.99–2.36) were more likely to reach the minimum requirements as per robotic training ‘reference center’ definition. We highlighted a nationwide lack of standardized training pathways, limited mentoring opportunities, and widespread dissatisfaction among robotic surgery trainees. These findings may guide robotic programs in recognizing unmet educational needs and implementing strategies to develop equitable, structured, and standardized curricula for robotic surgical training across the multiple robotic-assisted surgical specialties.

**Supplementary Information:**

The online version contains supplementary material available at https://doi.org/10.1007/s13304-026-02747-y.

## Introduction

Since the arrival of the first robotic platform in Italy in 1999, robotic surgery has undergone a significant evolution [1–3]. In 2024, a total of 168 institutions across the country hosted more than 200 robotic systems [4]. Increased surgical precision, accuracy, and visualization are the major advantages of robotic-assisted surgery (RAS), which translate into lower estimated blood loss (EBL), shorter hospital stay, faster recovery, and improved cosmesis [1, 5–8]. Accordingly, RAS adoption has increased across different specialties [9–12]. Urology, general surgery and gynecology encompass 80% of current robotic-assisted procedures, while the remaining 20% involve specialties with an emerging robotic experience, such as thoracic surgery, head and neck, orthopaedics and cardiac/vascular surgery [13].

Significant efforts have been made to eliminate inequalities in patient care and promote widespread access to robotic platforms for patients across different countries and regions in Europe [3]. However, equal attention was not given to reducing deficiencies in robotic surgical training by promoting standardized training curricula for medical students, residents, and young attendings. After an initial phase where RAS was the exclusive domain of expert attending surgeons, access to the robotic console is now expanding to both residents and junior attendings. Nonetheless, in most institutions, the breadth of surgical training is still dependent on both the availability and willingness of the attending surgeon to allow hands-on trainee mentoring, often resulting in wide differences in the preparation levels of graduating residents [14, 15]. Even when hands-on training is employed, it is generally accompanied by a lack of standardization and objective feedback, creating additional issues. Also, few institutions across Europe have adopted a standardized training curriculum for robotic surgery, such as Proficiency Based Progression (PBP), which relies on a step-by-step progression of predefined performance metrics and proficiency levels [16–18]. Not surprisingly, PBP has demonstrated higher performance outcomes and trainees’ satisfaction [19]. In particular for Italy, due to the extremely limited availability of standardized training programs and certified fellowships, the majority of residents and young attendings with an interest in robotic surgery tend to seek additional training abroad after completion of their residency training.

In this manuscript, we reported the results of a survey, developed with a modified-Delphi consensus, on robotic surgery adoption and training status conducted among residents and young attendings (i.e., < 5 years in practice) across different surgical specialties. Moreover, we sought to identify predictors linked with increased satisfaction with both the overall robotic surgical experience and with a higher exposure to RAS. The ultimate aim was to provide guidance for future programs as per improving and standardizing robotic training curricula across the country in all the different robotic-assisted surgical specialties involved.

## Materials and methods

### The survey and modified-Delphi consensus

The survey was developed through an already validated standardized methodology [20]. Four young surgeons with experience in robotic surgery and at least one international fellowship in a high-volume reference center for robotic surgery training (2 Urologists [FDG and VS], 1 General Surgeon [MA], 1 Gynecologist [SM]), all affiliated with the Italian Multispecialty Society of Young Surgeons (SPIGC), developed the original draft of the questionnaire. Subsequently, a panel of 15 young surgeons (4 Urologists, 3 General Surgeons, 2 Gynecologists, 2 Plastic Surgeons, 1 Thoracic Surgeon, 1 Vascular surgeon, 1 orthopaedic, and 1 ENT surgeon) with experience in robotic surgery from 4 different countries (10 from Italy, 3 from the United Kingdom, 1 from Belgium, and 1 from France), reviewed and validated the questionnaire with a modified Delphi process [21]. The video conference for the Delphi consensus took place in December 2024.

The survey structure is summarized in supplementary Table 1. The survey comprises 4 modules (M1–M4), with a total of 41 multiple-choice or open-ended questions, organized as follows: M1 (Q1–Q11), Demographic data; M2 (Q12–Q23), Robotic surgery prevalence across surgical institutions; M3 (Q24–Q35), Trainee involvement in robotic-assisted procedures; M4 (Q36–Q41), Training curricula and expertise. Most of the answer options were formatted as a five-choice Likert-type scale.

### Study population and survey participants

After validation, the final questionnaire was developed with Google Forms platform and distributed via e-mail and social networks. Particularly, survey distribution, answering of query-related questions and confirmation of final authorship were performed via a dedicated e-mail account (i.e., *surveyspigclazio@gmail.com*). Moreover, survey promotion was carried out via the mailing list of Italian surgical residents and trainees, instant messaging platforms (such as Telegram and Whatsapp), the official SPIGC website and account, as well as various social media channels. Residents, fellows, PhD students, young attendings and assistant professors, in Urology, general surgery, Obstetrics and Gynecology (OB&GYN), Plastic Surgery, Thoracic Surgery, ENT Surgery, Orthopaedics, Neurosurgery, Maxillofacial Surgery and Vascular surgery were included. Exclusion criteria included inability to complete the survey due to literacy or language barriers and other potential conflicts of interest. Survey participants were informed about the survey’s purpose, its expected completion time, and the aims of the investigators. Anonymity was guaranteed by the study design and co-authorship in the SPIGC collaborative group was offered on a voluntary basis. No payment for participation was offered. Answers were recorded directly on Google Forms between Feb and Apr 2025. This study was reported in accordance with the guidelines set by the American Association for Public Opinion Research, as well as the CHEcklist for Reporting Results of Internet E-Surveys (CHERRIES) framework [22].

### Definition of satisfaction and reference robotic training center

To assess the overall satisfaction with robotic surgery exposure and training, a composite variable (from 2 to 10) was created, by computing the cumulative responses from the two distinct satisfaction-related questions - “Are you satisfied with the overall training in Robotic Surgery at your center?” and “Are you satisfied with your involvement during robotic surgery procedures?” - each rated on an ordinal five-point Likert-type scale (1–5). The strong positive correlation between the two items (Pearson correlation coefficient *r* = 0.772) and high internal consistency reliability (Cronbach’s Alpha = 0.887) confirmed the methodological validity of this approach.

Then, a composite binary variable (the “bifecta”, YES vs. NO), was created, taking into account both pre-clinical factors (availability of the dual console, availability of simulation software, and scheduled didactic lectures) and clinical factors (number of monthly procedures performed as a console surgeon or as a bed-side assistant by a trainee) that are largely associated with high-quality robotic training. To achieve the “bifecta” the following two conditions had to be simultaneously met: (1) At least 2 out of 3 preclinical factors, amongst dual console, simulation software, and scheduled lectures and (2) At least 1 procedure monthly as console surgeon or 3 as bed-side assistant performed by the trainee. These thresholds were defined a priori by consensus and represent an exploratory construct rather than a previously validated training metric. Subsequently, a multivariable binary logistic regression model was built, to identify variables influencing bifecta achievement.

### Statistical analysis

All final answers were extracted and some questions were simplified for statistical analysis. Descriptive statistics were adopted to summarize survey results. Numbers and percentages were used to express categorical variables and continuous data was summarized adopting median, mean, interquartile range (IQR) and standard deviation (SD) when appropriate. For categorical variables, the existence of a significant association was evaluated with the Pearson Chi-square test or the Fisher’s exact test. Regarding quantitative data and pairwise intergroup comparisons of variables, associations were tested using a Mann-Whitney test or ANOVA one-way test. Subsequently, the impact of predetermined relevant variables on trainees’ satisfaction on robotic training, was explored adopting multivariable ordinal logistic regression modeling. Finally, multivariable binary logistic regression was implemented to assess factors associated with high-quality robotic training. Statistical analysis was performed with Stata version 18.1 (Stata Corporation, College Station, TX, USA) and SPSS version 27.0 (IBM SPSS Statistics for Windows, Version 27.0. Armonk, NY, USA: IBM Corp).

## Results

### Study cohort characteristics

General demographic and professional characteristics of the study cohort are reported in Table 1. A total of 735 (*n* = 467 [63.5%] males, *n* = 266 [36.2%] females and *n* = 2 [0.3%] non-disclosed) answered the questionnaire and were included in the final analysis. The majority of the participants were from the central (*n* = 353, 47.9%) part of the country, with *n* = 192 (26.1%) and *n* = 190 (26%) participants from institutions in the south and north, respectively. More than half of the included young physicians worked or trained in a University Hospital (*n* = 395, 53.7%), while participants from completely private institutions were the least represented (*n* = 5, 0.7%). There were 383 residents (52.1%), 25 PhD students (3.4%), 292 young attendings (39.7%), and 35 Post-doc research fellows (4.8%). 70.4% (*n* = 517) of the study cohort worked or trained in either Urology (*n* = 191) or General Surgery (*n* = 326).

**Table 1:** Baseline characteristics of the study cohort

Age, median (IQR)	Total = 735
	32 (29–35)
*Gender*	
Male	467 (63.5%)
Female	266 (36.2%)
Not answered	2 (0.3%)
*Region*	
Lazio	299 (40.7%)
Campania	137 (18.6%)
Puglia	23 (3.1%)
Lombardia	97 (13.2%)
Calabria	10 (1.4%)
Abruzzo	4 (0.5%)
Liguria	15 (2%)
Piemonte	24 (3.3%)
Friuli Venezia Giulia	9 (1.2%)
Emilia Romagna	16 (2.2%)
Toscana	39 (5.3%)
Veneto	28 (3.8%)
Sicilia	14 (1.9%)
Marche	2 (0.3%)
Basilicata	3 (0.4%)
Sardegna	4 (0.5%)
Umbria	9 (1.2%)
Valle d’Aosta	1 (0.1%)
Molise	1 (0.1%)
*Macro-regions*	
South	192 (26.1%)
Center	353 (47.9%)
North	190 (26%)
*Hospital/company type*	
AOU (University Hospital)	395 (53.7%)
AO (Public Hospital)	93 (12.7%)
ASL (Local Health Authority)	33 (4.5%)
IRCCS (Research Hospital)	134 (18.2%)
SSP (Private Hospital)	5 (0.7%)
SSP-accreditata (Partially public)	67 (9.1%)
AUSL (Local Health Unit)	8 (1.1%)
*Role*	
Resident	383 (52.1%)
PhD Student	25 (3.4%)
Young Attending	292 (39.7%)
Post-doc Research Fellow	35 (4.8%)
*PGY*	
1	38 (5.2%)
2	42 (5.7%)
3	77 (10.5%)
4	115 (15.6%)
5	111 (15.1%)
*Specialty*	
General Surgery	326 (44.4%)
Urology	191 (26.0%)
Vascular surgery	11 (1.5%)
Maxillofacial surgery	17 (2.3%)
Thoracic Surgery	60 (8.2%)
Orthopaedics	29 (3.9%)
OB&GYN	71 (9.7%)
Plastic Surgery	26 (3.5%)
Neurosurgery	2 (0.3%)
ENT	2 (0.3%)

IQR: Interquartile Range; AOU: University Hospital; ASL, AUSL and AO: Public non University Hospitals; IRCCS: Public Research Institution; SSP: Private institution; SSP-accreditata; Semi-private institution PhD: Doctor of Philosophy; PGY: Post-Graduate Year; ENT: Ear, Nose and Throat

### Robotic surgery exposure by surgical specialty

The results of selected relevant questions for robotic surgery training, exposure, and satisfaction are reported, stratified by specialty, in Table 2. 53.7% (*n* = 175) of participants in general surgery, 85.9% (*n* = 164) in Urology, 87.3% (*n* = 62) in OB&GYN, 70% (*n* = 42) in thoracic surgery and 60.9% (*n* = 53) in other specialties reported adequate tutoring offered by experienced surgeons (*p* < 0.001). The availability of a dual console, simulation software, or scheduled lectures were documented by 45.6% (*n* = 335), 58.2% (*n* = 428) and 23.4% (*n* = 172), respectively. In particular, a dual console and scheduled lectures were more frequent for OB&GYN (60.6% and 36.6%), while Urology had the highest percentage of reported simulation software availability (78.5%). However, only 55.1% (*n* = 405) of the total cohort would recommend their institution as a robotic surgery training center and 27.9% (*n* = 205) would consider changing from their current institution to a different reference center for robotic surgery. Urology and OB&GYN had the highest median satisfaction (from 1 to 5) with the overall robotic training at their institution (3 [3–4]) and general surgery had the lowest (2 [1–3]) (*p* < 0.001). Similar results were obtained when considering satisfaction with involvement during robotic-assisted procedures (*p* < 0.001). Accordingly, only 8.3% (*n* = 27) of participants for general surgery usually act as console surgeons during robotic procedures, compared to 17.8% (*n* = 34) and 18.3% (*n* = 13) for Urology and OB&GYN (*p* < 0.001), respectively. Also, *n* = 129 (17.6%) participants had experience in qualified reference centers for robotic surgery abroad. The majority (*n* = 77) reported greater clinical involvement in robotic procedures in the international center, compared to their original Italian institution. Finally, only *n* = 49 trainees for general surgery (15%), 79 for urology (41.4%), 27 for OB&GYN (38%), 12 for thoracic surgery (20%) and 15 for other specialties (17.2%) reported any kind of fellowship, master’s degree or post-specialty training program in robotic surgery to be available at their institution (*p* < 0.001).

**Table 2:** Survey results stratified by specialty

	TOtal	General surgery	Urology	Obs and gynae	Thoracic surgery	Other	*p*-value
Are you aware of the applications of Robotic Surgery in your specialty							
Yes	676 (92.0%)	303 (92.9%)	186 (97.4%)	70 (98.6%)	59 (98.3%)	58 (66.7%)	< 0.001
No	59 (8.0%)	23 (7.1%)	5 (2.6%)	1 (1.4%)	1 (1.7%)	29 (33.3%)	
Median interest in robotic surgery	5 (4–5)	5 (4–5)	5 (4–5)	4 (3–5)	5 (4–5)	4 (3–5)	-
Number of robotic-assisted procedures performed annually in your center							
0	74 (10.1%)	35 (10.7%)	8 (4.2%)	4 (5.6%)	1 (1.7%)	26 (29.9%)	< 0.001
< 100	171 (23.3%)	96 (29.4%)	14 (7.3%)	8 (11.3%)	22 (36.7%)	31 (35.6%)	
100–250	255 (34.7%)	111 (34.0%)	84 (44.0%)	21 (29.6%)	19 (31.7%)	20 (23.0%)	
250–400	144 (19.6%)	51 (15.6%)	60 (31.4%)	16 (22.5%)	11 (18.3%)	6 (6.9%)	
> 400	91 (12.4%)	33 (10.1%)	25 (13.1%)	22 (31.0%)	7 (11.7%)	4 (4.6%)	
Number of robots available	1 (1–2)	1 (1–2)	1 (1–2)	3 (1–3)	2 (1–3)	1 (0–2)	< 0.001
Number of experienced (> 50 procedures) surgeons	3 (2–5)	3 (2–5)	3 (2–4)	6 (2–10)	3 (1–4)	2 (0–5)	0.001
Are experienced surgeons offering adequate tutoring?							
No	239 (32.5%)	151 (46.3%)	27 (14.1%)	9 (12.7%)	18 (30.0%)	34 (39.1%)	< 0.001
Yes	496 (67.5%)	175 (53.7%)	164 (85.9%)	62 (87.3%)	42 (70.0%)	53 (60.9%)	
Is a double console available at your center?							
No	400 (54.4%)	195 (59.8%)	82 (42.9%)	28 (39.4%)	31 (51.7%)	64 (73.6%)	< 0.001
Yes	335 (45.6%)	131 (40.2%)	109 (57.1%)	43 (60.6%)	29 (48.3%)	23 (26.4%)	
Are simulation software available and usable?							
No	307 (41.8%)	154 (47.2%)	41 (21.5%)	40 (56.3%)	20 (33.3%)	52 (59.8%)	< 0.001
Yes	428 (58.2%)	172 (52.8%)	150 (78.5%)	31 (43.7%)	40 (66.7%)	35 (40.2%)	
Are there scheduled lectures on the use of robotic platforms, robotic perioperative assistance, and/or surgical techniques?							
No	563 (76.6%)	266 (81.6%)	140 (73.3%)	45 (63.4%)	46 (76.7%)	66 (75.9%)	0.009
Yes	172 (23.4%)	60 (18.4%)	51 (26.7%)	26 (36.6%)	14 (23.3%)	21 (24.1%)	
Would you recommend your institution as a robotic surgery training center							
No	330 (44.9%)	201 (61.7%)	46 (24.1%)	12 (16.9%)	25 (41.7%)	46 (52.9%)	< 0.001
Yes	405 (55.1%)	125 (38.3%)	145 (75.9%)	59 (83.1%)	35 (58.3%)	41 (47.1%)	
Would you consider changing your residency program and/or training center to join a reference center for Robotic Surgery training?							
No	530 (72.1%)	199 (61.0%)	142 (74.3%)	64 (90.1%)	53 (88.3%)	72 (82.8%)	< 0.001
Yes	205 (27.9%)	127 (39.0%)	49 (25.7%)	7 (9.9%)	7 (11.7%)	15 (17.2%)	
Are you satisfied with the overall training in Robotic Surgery at your center?	3 (2–4)	2 (1–3)	3 (3–4)	3 (3–4)	3 (2–4)	3 (1–4)	< 0.001
Are you satisfied with the overall training in Robotic Surgery at your center?							< 0.001
1	163 (22.2%)	100 (30.7%)	16 (8.4%)	9 (12.7%)	10 (16.7%)	28 (32.2%)	
2	141 (19.2%)	85 (26.1%)	26 (13.6%)	8 (11.3%)	8 (13.3%)	14 (16.1%)	
3	194 (26.4%)	70 (21.5%)	62 (32.5%)	25 (35.2%)	15 (25%)	22 (25.3%)	
4	150 (20.4%)	50 (15.3%)	53 (27.7%)	17 (23.9%)	14 (23.3%)	16 (18.4%)	
5	87 (11.8%)	21 (6.4%)	34 (17.8%)	12 (16.9%)	13 (21.7%)	7 (8%)	
During a robotic surgery procedure at your center, who performs the different surgical steps as the primary console surgeon?							
Expert surgeon only	453 (61.6%)	235 (72.1%)	81 (42.4%)	45 (63.4%)	36 (60.0%)	56 (64.4%)	< 0.001
Expert surgeon + junior staff member	184 (25.0%)	64 (19.6%)	74 (38.7%)	21 (29.6%)	11 (18.3%)	14 (16.1%)	
Expert surgeon + junior staff member or resident	98 (13.3%)	27 (8.3%)	36 (18.8%)	5 (7.0%)	13 (21.7%)	17 (19.5%)	
What is the percentage of robotic surgeries performed by residents/junior staff as primary console surgeons compared to expert surgeons?							
None	328 (44.6%)	191 (58.6%)	44 (23.0%)	20 (28.2%)	29 (48.3%)	44 (50.6%)	< 0.001
10%	208 (28.3%)	85 (26.1%)	63 (33.0%)	21 (29.6%)	16 (26.7%)	23 (26.4%)	
20–30%	133 (18.1%)	34 (10.4%)	56 (29.3%)	20 (28.2%)	11 (18.3%)	12 (13.8%)	
40–50%	52 (7.1%)	14 (4.3%)	22 (11.5%)	7 (9.9%)	2 (3.3%)	7 (8.0%)	
60–80%	11 (1.5%)	1 (0.3%)	5 (2.6%)	3 (4.2%)	1 (1.7%)	1 (1.1%)	
90–100%	3 (0.4%)	1 (0.3%)	1 (0.5%)	0 (0.0%)	1 (1.7%)	0 (0.0%)	
Do you think robotic surgery should be reserved exclusively for expert surgeons?							
No	626 (85.2%)	295 (90.5%)	162 (84.8%)	53 (74.6%)	53 (88.3%)	63 (72.4%)	< 0.001
Yes	109 (14.8%)	31 (9.5%)	29 (15.2%)	18 (25.4%)	7 (11.7%)	24 (27.6%)	
Who usually acts as the scrubbed bedside assistant during robotic surgery?							< 0.001
Expert surgeon	193 (26.3%)	99 (30.4%)	25 (13.1%)	10 (14.1%)	24 (40.0%)	35 (40.2%)	
Resident	238 (32.4%)	49 (15.0%)	108 (56.5%)	42 (59.2%)	12 (20.0%)	27 (31.0%)	
Junior staff member/PhD student/Research fellow	304 (41.4%)	178 (54.6%)	58 (30.4%)	19 (26.8%)	24 (40.0%)	25 (28.7%)	
What is your main role during robotic surgery procedures?							< 0.001
Console surgeon	86 (11.7%)	27 (8.3%)	34 (17.8%)	13 (18.3%)	5 (8.3%)	7 (8.0%)	
Bedside assistant	358 (48.7%)	162 (49.7%)	104 (54.5%)	35 (49.3%)	35 (58.3%)	22 (25.3%)	
Observer	291 (39.6%)	137 (42.0%)	53 (27.7%)	23 (32.4%)	20 (33.3%)	58 (66.7%)	
Do you feel this is affecting your training path?							< 0.001
No	86 (11.7%)	24 (7.4%)	16 (8.4%)	10 (14.1%)	4 (6.7%)	32 (36.8%)	
Yes	649 (88.3%)	302 (92.6%)	175 (91.6%)	61 (85.9%)	56 (93.3%)	55 (63.2%)	
How many procedures or parts of procedures do you perform on average per month as the primary console surgeon?	0 (0–1)	0 (0–0)	0 (0–2)	0 (0–2.5)	0 (0–1.25)	0 (0–0)	0.020
How many procedures or parts of procedures do you assist on average per month as a bedside assistant during robotic surgery?	2 (0–5)	1.5 (0–5)	4 (0–10)	2 (0–6)	3 (1–7.75)	0 (0–2)	< 0.001
Are you satisfied with your involvement during robotic surgery procedures?	3 (1–4)	2 (1–3)	3 (2–4)	3 (1–4)	3 (1–4)	2 (1–3)	< 0.001
Are you satisfied with your involvement during robotic surgery procedures?							
1	241 (32.8%)	135 (41.4%)	30 (15.7%)	18 (25.4%)	19 (31.7%)	39 (44.8%)	< 0.001
2	124 (16.9%)	64 (19.6%)	28 (14.7%)	13 (18.3%)	7 (11.7%)	12 (13.8%)	
3	177 (24.1%)	68 (20.9%)	58 (30.4%)	14 (19.7%)	15 (25.0%)	22 (25.3%)	
4	117 (15.9%)	39 (12.0%)	46 (24.1%)	16 (22.5%)	11 (18.3%)	5 (5.7%)	
5	76 (10.3%)	20 (6.1%)	29 (15.2%)	10 (14.1%)	8 (13.3%)	9 (10.3%)	
At which year of residency do you believe trainees should first be involved in robotic surgery training?							
Year 1–2	210 (28.6%)	96 (29.4%)	49 (25.7%)	24 (33.8%)	19 (31.7%)	22 (25.3%)	0.209
Year 3	382 (52.0%)	155 (47.5%)	111 (58.1%)	32 (45.1%)	33 (55.0%)	51 (58.6%)	
Year 4–5	143 (19.5%)	75 (23.0%)	31 (16.2%)	15 (21.1%)	8 (13.3%)	14 (16.1%)	
Have you ever had experience in qualified reference centers for Robotic Surgery in Italy?							0.195
No	532 (72.4%)	231 (70.9%)	138 (72.3%)	51 (71.8%)	40 (66.7%)	72 (82.8%)	
Yes	203 (27.6%)	95 (29.1%)	53 (27.7%)	20 (28.2%)	20 (33.3%)	15 (17.2%)	
For how long?							0.014
6 months	84 (11.4%)	46 (14.1%)	18 (9.4%)	3 (4.2%)	9 (15.0%)	8 (9.2%)	
1 year	45 (6.1%)	21 (6.4%)	16 (8.4%)	2 (2.8%)	3 (5.0%)	3 (3.4%)	
> 1 year	74 (10.1%)	28 (8.6%)	19 (9.9%)	15 (21.1%)	8 (13.3%)	4 (4.6%)	
Have you ever had experience in qualified reference centers for Robotic Surgery abroad?							0.123
No	606 (82.4%)	260 (79.8%)	157 (82.2%)	62 (87.3%)	48 (80.0%)	79 (90.8%)	
Yes	129 (17.6%)	66 (20.2%)	34 (17.8%)	9 (12.7%)	12 (20.0%)	8 (9.2%)	
For how long?							0.003
6 months	67 (9.1%)	41 (12.6%)	7 (3.7%)	5 (7.0%)	9 (15.0%)	5 (5.7%)	
1 year	40 (5.4%)	16 (4.9%)	18 (9.4%)	1 (1.4%)	2 (3.3%)	3 (3.4%)	
> 1 year	22 (3.0%)	9 (2.8%)	9 (4.7%)	3 (4.2%)	1 (1.7%)	0 (0.0%)	
The clinical involvement of the Resident/Fellow in Robotic Surgery compared to your Italian reference center is:							0.063
Less	13 (1.8%)	6 (1.8%)	1 (0.5%)	3 (4.2%)	2 (3.3%)	1 (1.1%)	
Comparable	39 (5.3%)	14 (4.3%)	13 (6.8%)	3 (4.2%)	5 (8.3%)	4 (4.6%)	
Greater	77 (10.5%)	46 (14.1%)	20 (10.5%)	3 (4.2%)	5 (8.3%)	3 (3.4%)	
Does your center offer a fellowship or a post-specialty training program in robotic surgery?							< 0.001
No	553 (75.2%)	277 (85.0%)	112 (58.6%)	44 (62.0%)	48 (80.0%)	72 (82.8%)	
Yes	182 (24.8%)	49 (15.0%)	79 (41.4%)	27 (38.0%)	12 (20.0%)	15 (17.2%)	
Do you think industry partners should play an active role in the training of residents, junior doctors, PhD candidates, or research fellows in robotic surgery?							0.153
No	78 (10.6%)	31 (9.5%)	27 (14.1%)	4 (5.6%)	4 (6.7%)	12 (13.8%)	
Yes	657 (89.4%)	295 (90.5%)	164 (85.9%)	67 (94.4%)	56 (93.3%)	75 (86.2%)	
Would you like to deepen your knowledge in Robotic Surgery at another center in Italy?							< 0.001
No	280 (38.1%)	80 (24.5%)	81 (42.4%)	49 (69.0%)	25 (41.7%)	45 (51.7%)	
Yes	455 (61.9%)	246 (75.5%)	110 (57.6%)	22 (31.0%)	35 (58.3%)	42 (48.3%)	
Would you like to deepen your knowledge in Robotic Surgery in another country?							0.043
No	287 (39.0%)	117 (35.9%)	77 (40.3%)	39 (54.9%)	20 (33.3%)	34 (39.1%)	
Yes	448 (61.0%)	209 (64.1%)	114 (59.7%)	32 (45.1%)	40 (66.7%)	53 (60.9%)	

### Variables affecting satisfaction with robotic surgery training and exposure

The highest median overall satisfaction was reported by Urology (6 [5–8]), followed by Thoracic Surgery and OB&GYN (6 [4–8]), other specialties (5 [2–6]) and then General Surgery (4 [2–6]) (*p* < 0.001) (Table 3). Also, the results of the ordinal multivariable logistic regression exploring the influence of predetermined variables on the overall satisfaction score is reported in Table 4. An increasing number of robotic-assisted procedures performed annually correlated with a higher overall satisfaction (adjusted Odds Ratio [aOR]: 9.69 [5.62–16.70], *p* < 0.001 for > 400 procedures). The availability of simulation software and scheduled lectures were found to have an independent significant positive correlation with overall satisfaction (aOR:1.72 [1.22–2.44], *p* = 0.002 and aOR: 1.72 [1.16–2.56], *p* = 0.007 respectively for simulation software and scheduled lectures). As expected, an increasing percentage of robotic procedures performed by residents or junior staff members positively correlates with higher overall satisfaction (aOR:33.75 [2.74–416.43], *p* = 0.006 for 90–100%). Conversely, acting primarily as an observer during robotic procedures significantly reduced the overall satisfaction (aOR:0.24 [0.16–0.36]). Ultimately, at multivariate analysis, the specialty area did not significantly influence the overall satisfaction level.

**Table 3:** Overall Satisfaction by specialty

Satisfaction by specialty		Satisfaction total									*p* value	
Specialty	Median	2	3	4	5	6	7	8	9	10		OR
Urology (Ref.)	6 (5–8)	14 (7.3%)	10 (5.2%)	18 (9.4%)	18 (9.4%)	39 (20.4%)	22 (11.5%)	34 (17.8%)	15 (7.9%)	21 (11%)	**< 0.001**	1.00
Other	5 (2–6)	27 (31%)	6 (6.9%)	10 (11.5%)	6 (6.9%)	18 (20.7%)	6 (6.9%)	6 (6.9%)	5 (5.7%)	3 (3.4%)		0.30
General Surgery	4 (2–6)	90 (27.4%)	41 (12.6%)	42 (12.9%)	37 (11.3%)	46 (14.1%)	21 (6.4%)	24 (7.4%)	13 (4%)	12 (3.7%)		0.28
Thoracic Surgery	6 (4–8)	9 (15%)	3 (5%)	7 (11.7%)	8 (13.3%)	8 (13.3%)	7 (11.7%)	6 (10%)	6 (10%)	6 (10%)		0.70
OB&GYN	6 (4–8)	8 (11.3%)	5 (7%)	8 (11.3%)	8 (11.3%)	14 (19.7%)	4 (5.6%)	12 (16.9%)	3 (4.2%)	9 (12.7%)		0.76

**Table 4:** Multivariable ordinal regression analysis for overall satisfaction

Variable	aOR	95% CI	*p* value
Number of robotic-assisted procedures performed annually in your center	–	–	–
0 (Ref.)	1.00	–	–
< 100	4.00	2.52–6.35	0.010
100–250	8.92	5.87–13.55	< 0.001
250–400	7.69	3.06–19.31	< 0.001
> 400	9.69	5.62–16.70	< 0.001
Is a double console available at your center?	–	–	–
No (Ref.)	1.00	–	–
Yes	0.75	0.54–1.06	0.102
Are simulation software available and usable?	–	–	–
No (Ref.)	1.00	–	–
Yes	1.72	1.22–2.44	0.002
Are there scheduled lectures on robotic platforms, perioperative assistance, and/or surgical techniques?	–	–	–
No (Ref.)	1.00	–	–
Yes	1.72	1.16–2.56	0.007
During a robotic surgery procedure, who performs the different surgical steps as the primary console surgeon?	–	–	–
Expert surgeon only (Ref.)	1.00	–	–
Expert surgeon + junior staff member	0.88	0.58–1.33	0.538
Expert surgeon + junior staff member or resident	2.00	1.18–3.40	0.010
What is the percentage of robotic surgeries performed by resident/junior staff as primary console surgeons compared to expert surgeons?	–	–	–
None (Ref.)	1.00	–	–
10%	3.06	2.02–4.62	< 0.001
20–30%	5.96	3.44–10.32	< 0.001
40–50%	7.59	3.56–16.18	< 0.001
60–80%	6.40	1.56–26.24	0.010
90–100%	33.75	2.74–416.43	0.006
What is your main role during robotic surgery procedures?	–	–	–
Bedside Assistant (Ref.)	1.00	–	–
Console surgeon	2.43	1.29–4.57	0.006
Observer	0.24	0.16–0.36	< 0.001
Have you ever had experience in qualified reference centers for Robotic Surgery in Italy?	–	–	–
No (Ref.)	1.00	–	–
Yes	0.90	0.63–1.28	0.568
Have you ever had experience in qualified reference centers for Robotic Surgery abroad?	–	–	–
No (Ref.)	1.00	–	–
Yes	1.22	0.82–1.82	0.328
Does your center offer a fellowship or a post-specialty training program in robotic surgery?	–	–	–
No (Ref.)	1.00	–	–
Yes	1.41	0.95–2.08	0.083
Specialty	–	–	–
Urology (Ref.)	1.00		
Other	1.92	0.93–4.88	0.064
General Surgery	1.08	0.65–1.56	0.748
Thoracic Surgery	1.87	0.78–4.07	0.165
OB&GYN	1.52	0.83–2.82	0.207

aOR: Adjusted Odds Ratio; 95% CI: 95% Confidence Interval

### Variables defining a Reference Center for Robotic-assisted Surgical Training

**Fig. 1:**
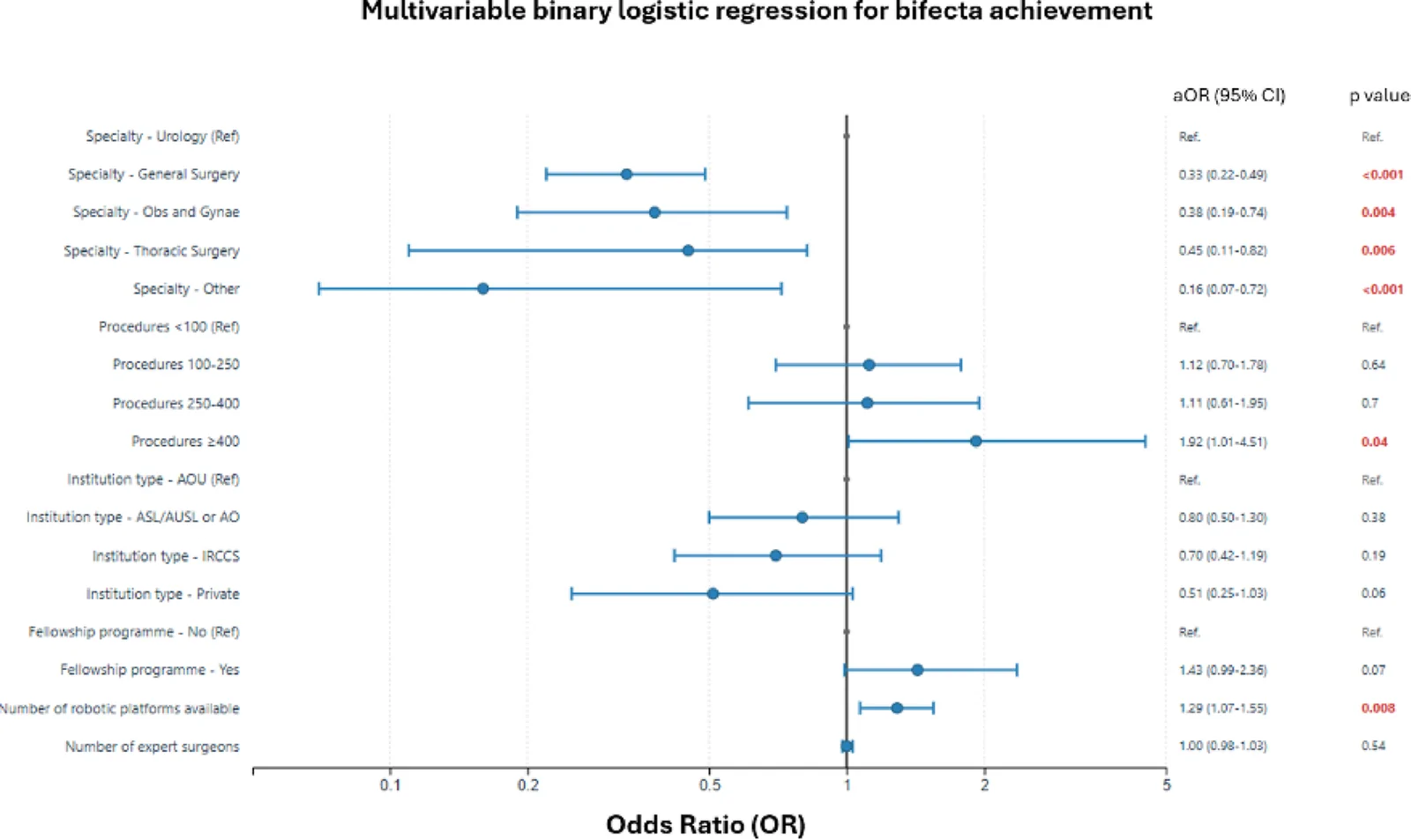
Forest Plot depicting the results of the multivariate regression analysis for bifecta achievement. aOR: Adjusted Odds Ratio; 95% CI: 95% Confidence Interval; AOU: University Hospital; ASL, AUSL and AO: Public non University Hospitals; IRCCS: Public Research Institution

The results of multivariable logistic regression exploring the impact of relevant variables on bifecta achievement are reported in Table 5 and visually depicted in Fig. 1. When compared to Urology, training in other specialties decreased the probability of reaching the bifecta (aOR: 0.33, 95% CI 0.22–0.49 for general surgery, *p* < 0.001; aOR: 0.38, 95% CI 0.19–0.74, *p* = 0.004 for OB&GYN; aOR: 0.45, 95% CI 0.11–0.82, *p* = 0.006 for thoracic surgery). A center where > 400 procedures were performed annually was more likely to meet the criteria for the bifecta (aOR: 1.92, 95% CI 1.01–4.51, *p* = 0.040). Having a fellowship program in robotic surgery also increased the likelihood of achieving the bifecta, but the difference was slightly over the level of significance (aOR: 1.43, 95% CI 0.99–2.36, *p* = 0.07). Finally, an increasing number of robotic platforms available positively correlated with bifecta achievement (aOR: 1.29, 95% CI 1.07–1.55, *p* = 0.008).

**Table 5:** Multivariable Binary regression analysis for bifecta achievement

Specialty	aOR	95% CI	*p* value
Urology	Ref.		
General Surgery	0.33	0.22–0.49	< 0.001
OB&GYN	0.38	0.19–0.74	0.004
Thoracic Surgery	0.45	0.11–0.82	0.006
Other	0.16	0.07–0.72	< 0.001
Number of robotic assisted procedures performed annually in the center			
< 100	Ref.		
100–250	1.12	0.70–1.78	0.642
250–400	1.11	0.6–1.95	0.702
> 400	1.92	1.01–4.51	0.040
Institution type			
University Hospital	Ref.		
Public Hospital or Local Health Authority	0.80	0.50–1.30	0.378
Research Hospital	0.70	0.42–1.19	0.189
Private	0.51	0.25–1.03	0.061
Fellowship programme at the center			
No	Ref.		
Yes	1.43	0.99–2.36	0.072
Number of robotic platforms available	1.29	1.07–1.55	0.008
Number of expert surgeons	1.00	0.98–1.03	0.544

aOR: Adjusted Odds Ratio; 95% CI: 95% Confidence Interval; AOU: University Hospital; ASL, AUSL and AO: Public non University Hospitals; IRCCS: Public Research Institution

## Discussion

The evaluation of the outcomes of RAS has been a main focus of surgical research in the last 20 years. Between 2010 and 2017, the rate of RAS performed in the USA increased by 2460% [23], with a similar trend observed in European institutions. A nationwide real-life analysis on nearly a million patients in Germany demonstrated an exponential increase in the number of patients undergoing RAS in the last two decades, from 14 cases in 2005 to 25,665 cases in 2021 [24]. Particularly, two thirds of Radical Prostatectomies (RP) and roughly 50% of Partial Nephrectomies (PNs) were being performed robotically by 2021. Despite this, the development of robotic training advanced at markedly different rates between European and American institutions. A recent survey performed on surgical trainees across different institutions in the USA, UK, and Ireland, found the percentage of trainees who had performed 15 or more cases as the console surgeon was 91.18% for US trainees, compared to only 3.44% for UK and 16.67% for Irish trainees (*p* < 0.00001) [23]. As such, not surprisingly, satisfaction with robotic training was significantly higher amongst US trainees. As with the American experience, it is becoming clear that having a standardized training program is necessary in order to minimize the impact of the learning curve on both outcomes and operative efficiency [25]. A structured training curriculum should include lectures, simulator practice, dry labs, and cadaver/animal labs. In line with the concept of PBP, access to real-life patients should be granted only to those trainees who demonstrated proficiency on previous steps of the training program [26–28].

In our present study, we reported the results of a survey conducted among 735 young Italian physicians at different stages in their surgical training, in order to describe current status and expectations of robotic training across our nation. Our results depict a scenario of limited exposure to both clinical and preclinical robotic training in our Italian institutions. Only 25% of the participants reported an active role as robotic console surgeon offered to surgical trainees and, when only residents were factored in, the percentage dropped to 13.3%. The great majority of the participants felt robotic surgery should not be reserved for expert surgeons alone (85.2%) and that the lack of exposure to robotic surgery was negatively affecting their training (88.3%).

Results from our analysis confirm the differences in our training system when compared to that of other countries, such as the USA. In fact, while a 2015 survey of American general surgery residents reported that only 18% had any exposure to the robotic console, by 2023 that figure had risen to 75%, with dual-console systems available in 85% of training centers [29, 30]. Conversely, a 2024 pan-European survey on the state of robotic training in gastrointestinal surgery involving 1045 respondents from 38 different countries revealed that more than half of the surgical trainees had no exposure to robotic surgery [31]. Of those few who did have robotic access, 48.2% had performed zero cases as console surgeons (13% of the total trainees), highlighting a generalized lack of standardized training opportunities across European institutions.

Our results also reveal significant discrepancies across different surgical specialties. In particular, specialties where robotic surgery has been in regular use for the longest period of time, such as Urology and Gynecology, appear to be more aware that adequate robotic training is a major need. As such, in those specialties with emerging robotic adoption (thoracic surgery, ENT, maxillofacial etc.) and where surgeons are more likely to be in the initial phases of their learning curve, the focus on surgical trainees appears to be de-emphasized. However, that being said, emerging robotic specialties should benefit from developing sophisticated robotic training curricula and view the limited attention previously given to surgical trainees by their predecessors as a cautionary example. Not surprisingly, when evaluating overall satisfaction with robotic training, surgical specialty was no longer significant on multivariate analysis, suggesting that the other variables (such as an active role during robotic cases, the access to robotic simulators and scheduled lectures or the case volume of the training center) play a more pivotal role in the perceived adequacy of the training program.

Finally, using both structural (availability of dual console, simulation software, and lectures) and clinical (active participation during robotic-assisted cases) factors, we created a variable which identifies the minimum requirements for an institution to be defined as a reference center for robotic surgery training. We found that Urologic centers were significantly more likely to meet the criteria for “bifecta” achievement. Moreover, the likelihood of achieving the bifecta was higher for centers with a high volume of robotic cases, with multiple operative theaters, and a higher likelihood of a robotics fellowship program. Our goal was to offer a real-life picture of the current status of specialty and post-specialty training programs in Italy and possibly help young physicians with a definite interest in robotic surgery make an informed decision for their next step in surgical education. Our analysis also ultimately strives to guide specialty and post-specialty programs directors in recognizing unmet needs and opportunities for improvement, supporting strategic decisions for investing in the development of a standardized training curriculum.

We recognize several limitations of our study. Despite the high number of participants, the survey merely represents a snapshot in each of the regions and institutions included. Although we were able to capture responses from a broad spectrum of specialties, training levels, and institutions, the diversity in terms of macro-regions (nearly half from the center of the country), specialties (80.1% from general surgery, urology and gynaecology) and gender (only 36.2% of female participants) was relatively limited. Also, participation was obtained on a voluntary basis, which may have introduced a selection bias, since individuals with access to robotic surgery or strong opinions about it may have been more likely to participate. This is particularly true for those emerging specialties with initial robotic experience, where we obtained a relatively small representative sample, possibly influencing the results. In addition, while we used rational and statistical methods to create composite variables identifying subjective (satisfaction) and objective (reference center) determinants of the quality of robotic exposure, those variables were not validated. Finally, some estimates derived from smaller subgroups (e.g., highest case-volume and console-involvement categories) carry wide confidence intervals, and their values should be interpreted with caution.

## Conclusions

The present study provides a real-life snapshot of the current status of robotic surgery training in Italy. Results of our survey, obtained by young physicians at different levels of their post-graduate surgical training across multiple surgical specialties, might suggest a general lack of standardized training programs, insufficient mentoring, and trainee dissatisfaction, with significant discrepancies among different surgical specialties. Our study highlights the need for a nationwide consensus on a standardized robotic training curriculum, ensuring broad diffusion and incorporating strategies to both overcome geographical obstacles and promote equitable access.

## Supplementary Information

Below is the link to the electronic supplementary material.

Supplementary Material 1

Supplementary Material 2
