# Supplementary material for: Robotic surgery across surgical specialties: an Italian survey on implementation, training, and satisfaction levels among trainees and young attendings: on behalf of the Italian Multispecialty Society of Young Surgeons (SPIGC)
Source: Updates Surg. 2026 Sep 7;78(7):2815–35. doi: 10.1007/s13304-026-02747-y (PMC13751746; doi:10.1007/s13304-026-02747-y)
Supplement: Supplementary file 1 [file 13304_2026_2747_MOESM2_ESM.pdf]

NAME	SURNAME	AFFILIATION	ORCID
Marco	Abate	Università degli studi di napoli Federico II	
Marcello	Abbattista	Chirurgia plastica del Policlinico di Bari	
Roberto	Acanfora	Policlinico Umberto I	
Caterina	Accardo	Abdominal Transplants Unit, ISMETT - UPMC, Palermo	
Stefano	Agnesi	Department of General Surgery, Policlinico di Monza, Via Carlo Amati 111, 20900 Monza MB, Italy	0000-0002-7472-2669
Alberto	Aiolfi	IRCCS ospedale Galeazzi-Sant'Ambrogio	
Nahia	Akhras	Sapienza	
Simone	Albisinni	Dipartimento di Scienze Chirurgiche, Università Tor Vergata	
Maria Vittoria	Alesi	Dipartimento di Scienze per la Salute della Donna e del Bambino e di Sanità Pubblica, Fondazione Policlinico Universitario A. Gemelli, IRCCS, UOC Ginecologia Oncologica, Rome, Italy.	0009-0003-2562-3829
Federico	Alviani	Policlinico Umberto I Univeristà di Roma "La Sapienza"	0009-0005-3611-1559
Antonio	Alvigi	Università di Napoli Federico II	0009-0006-4508-3454
Lavinia	Amato	Struttura complessa di Chirurgia Generale Polo Ospedaliero di Città di Castello USL Umbria1	0009000707308656
Chiara	Amatucci	ASL Roma 6	
Luca	Ambrosio	Operative Research Unit of Orthopaedic and Trauma Surgery, Fondazione Policlinico Universitario Campus Bio-Medico, Rome, Italy.	0000-0003-2424-1274
Ugo	Amicuzi	Research Unit of Orthopaedic and Trauma Surgery, Deparmental Faculty of Medicine and Surgery, Università Campus Bio-Medico di Roma, Rome, Italy	
Sara	Ammar	Fondazione Policlinico A. Gemelli IRCSS Roma	0009-0008-1823-7483
Michele	Ammendola	Department of Experimental and Clinical Medicine, "Magna Graecia" University Medical School, "Renato Dulbecco" University Hospital, Digestive Surgery Unit	0000-0001-8043-6100
Carla	Ammendolia	Università degli Studi Roma La Sapienza- Policlinico Umberto I	
Giorgio	Ammerata	UOC Chirurgia Generale, P.O. Di Venere. Bari	0000-0001-5485-5221
Carla	Andaloro	Azienda ospedaliera di Padova	
Eleonora	Andreucci	Azienda ospedaliera senese chirurgia generale a indirizzo oncologico	
Pasquale	Angellotto	Neuroscienze Federico II	
Bianca	Angelone	Universitá degli studi di Genova	
Alfredo	Annicchiarico	Department of Surgery, Azienda Sanitaria Locale di Parma, Vaio Hospital, Fidenza, Italy	0000-0003-3882-776X
Pietro	Anoldo	DAI di Chirurgia Generale, dei Trapianti e Gastroenterologia, UOC di Chirurgia Endoscopica, AOU Federico II di Napoli	0000-0001-6727-2973
Luca	Antonelli	Dipartimento di Urologia, Policlinico Umberto I, Roma, Italy	0000-0001-6067-5497
Amedeo	Antonelli	Dipartimento Scienze Chirurgiche, Università degli studi di Roma Tor Vergata	0000-0002-5773-6962
Alice	Antonioni	Università La Sapienza di Roma	
Michele	Antonucci	UOSD Urologia Robotica e mininvasiva, policlinico tor vergata roma	
Adriana Ionelia	Apostol	Dipartimento della Salute della Donna e del Bambino, Policlinico Universitario Agostino Gemelli, IRCCS Roma	
Vittorio	Aprile	Unità operativa complessa di chirurgia toracica, dipartimento cardio toraco vascolare, azienda ospedaliero-universitaria di Pisa, aoup, pisa	0009-0008-4614-9261
Martina	Arcieri	Clinic of Obstetrics and Gynecology, "Santa Maria della Misericordia" University Hospital, Azienda Sanitaria Universitaria Friuli Centrale, Udine, Italy	0000-0002-8257-0618
Francesco Leone	Arcieri	Università Sapienza di Roma - Dipartimento di Chirurgia	0009-0003-0626-8673
Davide	Arrigo	Fondazione Policlinico Universitario Agostino Gemelli IRCCS - Università Cattolica del Sacro Cuore	0009-0001-2939-9129
Mario	Ascione	Dipartimento di Sanità Pubblica, Università degli studi di Napoli Federico II	0000-0002-7845-1149
Vincenzo	Asero	La Sapienza Università di Roma	0000-0001-5293-9253
Donatella	Aterno	specializzanda Policlinico Gemelli	
Miriam	Attalla El Halabieh	Fondazione Policlinico Agostino Gemelli	0009-0008-2007-0969
Giulia	Atzori	Chirurgia toracica, senologica e della tiroide, Ospedale regionale Umberto Parini	
Riccardo	Avantifiori	Policlinico Luigi di Liegro	
Pasquale	Avella	Department of Medicine and Health Science "V. Tiberio", Hepatobiliary and Pancreatic Surgery Unit, University of Molise	
Fulvia	Aymerito	Chirurgia generale, Humanitas Research Hospital	-
Ilham	Azenkoud	Dipartimento di medicina materno infantile e scienze urologiche, unità di ginecologia ed ostetricia, università la sapienza di Roma ospedale policlinico Umberto I	

Giulia	Bacchiocchi	Chirurgia Generale e Urgenza, Ospedale Civile di Baggiovara	0000-0002-9755-6229
Davide	Baiamonte	Azienda Ospedaliera Ospedali Riuniti "Villa Sofia - Cervello"	0000-0001-9152-4050
Ludovica	Balsamo	Dipartimento Cardio-Toraco-Vascolare. Chirurgia Toracica - Ospedale San Martino, Genova	
Raffaele	Balsamo	UOC UROLOGIA, OSPEDALE MONALDI, AZIENDA OSPEDALIERA DEI COLLI	
Andrea	Barberis	S.C. Chirurgia Generale ed Epatobiliopancreatica. E.O. Ospedali Galliera, Genova	0000-0003-0472-0120
Chiara	Barbieri	Università Cattolica del Sacro Cuore	0009-0008-7234-8993
Maria Elisabetta	Bariatti	Dipartimento di Chirurgia, SC Breast Unit ASST-settelaghi di Varese	0009-0000-7915-0551
Biagio	Barone	Department of Urology, Ospedale San Paolo, ASL NA1 Centro, 80125 Naples, Italy	0000-0003-4884-132X
Greta	Basile	La Sapienza	0009-0009-3054-327X
Giuseppe	Basile	Department of Urology, IRCCS San Raffaele Hospital	
Mattia	Basilico	Aurelia Hospital, Roma	
Massimiliano	Bassi	UOC Chirurgia Toracica, Sapienza University of Rome	
Sebastiano Angelo	Bastobe	Dipartimento di scienze chirurgiche, uoc di chirurgia toracica, università degli studi di roma tor vergata	0009-0007-7234-4805
Marco	Baviello	Asl Taranto	
Francesco	Belia	Fondazione Policlinico Universitario Campus Bio-Medico, Via Alvaro del Portillo, 200, 00128 Roma, Italy	0000-0002-8981-7898
Vittoria	Bellato	UOSD Chirurgia Mininvasiva e dell'Apparato Digerente, Policlinico Universitario Tor Vergata	0000-0003-0123-1700
Fabrizio	Bellavia	Urologia, Policlinico Agostino Gemelli, Università Cattolica del Sacro Cuore, Roma.	
Alessandro	Bello	Università di Napoli Federico II	
Elena	Belloni	Ospedale San Camillo, chirurgia generale, d'urgenza e delle nuove tecnologie	
Francesco	Bennardo	Department of Health Sciences, Magna Graecia University of Catanzaro	0000-0002-6528-2681
Ilaria	Benzoni	ASST Cremona	0000-0003-0312-3703
Lorenza	Beomonte Zobel	Chirurgia Mininvasiva e dell'Apparato Digerente, Dipartimento di Scienze Chirurgiche, Università e Policlinico di Roma Tor Vergata, Roma.	0000-0002-9414-3496
Giammauro	Berardi	San Camillo forlanini	
Giovanna	Berardi	Chirurgia generale oncologica minivasiva azienda universitaria Federico II NAPOLI	
Alessandro	Bergna	Department of General Surgery, Humanitas San Pio X-Milan	0000-0001-6101-3989
Arianna	Bernardotto	Sapienza	
Pietro	Bertoglio	IRCCS Azienda Ospedaliero Universitaria di Bologna	0000-0002-6267-0099
Andrea	Biancucci	UOC Chirurgia Generale e Oncologica, AO San Giovanni Addolorata, Roma	0000-0001-9097-1633
Beatrice	Biffoni	Policlinico A. Gemelli	
Gabriele	Bignante	Department of Oncology, Division of Urology, University of Turin, Candiolo Cancer Institute, Candiolo, Turin, Italy	0009-0008-4269-6663
Federica	Billeci	Università degli studi di Roma tor vergata	0000-0002-4749-5376
Cecilia	Bocchetti	Università la Sapienza- Azienda ospedaliera universitaria Sant'Andrea	
Guido	Bocchino	Ortopedia e Traumatologia - Università Cattolica del Sacro Cuore sede di Roma	0009-0000-4591-4015
Daniela	Bocchino	Chirurgia Generale e dei trapianti, Unità di Chirurgia endocrino-metabolica e senologia, Università Federico II di Napoli	
Giulia	Bonavina	Department of Obstetric and Ginecology, IRCCS Multimedica, Milano	
Alessandro	Bonis	Thoracic Surgery Unit, Department of Cardiac, Thoracic, Vascular Sciences and Public Health - DSCTV, University of Padova, 35128, Italy.	0000-0001-9422-0597
Viktoria	Borissova	Dipartimento di Scienze Odontostomatologiche e Maxillo Facciali 'La Sapienza' Universitá di Roma	0009-0003-4879-9210
Gabriele	Bormolini	Irccs Ospedale San Raffaele	0009-0007-6885-2903
Davide	Borra	Università degli Studi di Milano Statale	
Roberto	Borri	Università degli studi di Torino	
Giacomo	Borroni	Chirurgia Generale Oncologica Mininvasiva, ASST-Settelaghi, Varese	0000-0002-9391-1174
Andrea	Bottari	SOD CHIRURGIA D'urgenza, AOU Careggi	0000-0003-2784-5954
Francesco	Bottone	UOC Urologia Università degli Studi della Campania "Luigi Vanvitelli" DAI Medico Chirurgico di Alta Specialità	
Claudio	Brancelli	Università degli studi di Verona	0009-0003-2190-3786
Margherita	Brivio	Università di Milano	

Francesco	Brucchi	University of Milan	0000-0003-3191-4369
Valentina Maria	Brunelli	Urologia policlinico Umberto I	
Eleonora	Bruno	Sapienza Università di Roma - Policlinico Umberto I	0009-0000-1514-3440
Matteo	Bruno	Dipartimento di Ginecologia Oncologica, Policlinico Universitario Agostino Gemelli, IRCCS	0000-0003-4372-4220
Mattia	Bruschi	Istituto tumori Regina Elena	
Rosaria	Burzio	Aou Federico II Napoli	
Alberto	Busetto	Dipartimento di scienze cardio-toraco-vascolari e sanità pubblica, Università degli studi di Padova	0000-0001-5284-3227
Pietro	Calabrese	Clinical Medicine and Surgery Department, Naples “Federico II” University, AOU “Federico II” - Via S. Pansini 5, 80131, Naples, Italy	
Federica	Calabria	Università degli studi di Napoli Federico 2	
Fabrizia	Calabrò	Departement of Surgical, Medical, Molecular and Critical Care Pathology, University of Pisa, Pisa, Italy	
Andrea	Calandriello	AOU Federico II	
Alessandro	Calarco	Ospedale San Carlo di Nancy	0000-0001-6893-2179
Giuseppe	Calderone	Università di verona	0000-0003-0573-5826
Giacomo	Calini	Department of Medical and Surgical Sciences, Alma Mater Studiorum - University of Bologna, Bologna, Italy.	0000-0002-7460-9578
Armando	Calogero	Scienze biomediche avanzare federico 2	
Federica	Campolo	Fondazione Policlinico Agostino Gemelli	0000000339623064
Vittorio	Canale	Ospedale Cardinal Massaia UOC Urologia ASL Asti	
Giulia	Canali	Department of Medical Surgical Sciences and Translational Medicine, Sapienza University of Rome, Sant'Andrea Hospital, Via di Grottarossa, 1035-1039, 00189 Rome, Italy	0000-0002-6536-5444
Maria	Candida	UOC Chirurgia Generale Emergenziale AOU Policlinico Federico II Napoli	
Giuseppe	Candita	Sapienza università di Roma	
Rocco	Cangiano	Dipartimento Cardio-Toraco-Vascolare e chirurgia dei trapianti d'organo - Policlinico Umberto I - "Sapienza" Università di Roma	
Gianluca	Canu	UOC Chirurgia Toracica - Dipartimento di Scienze Cardio-Toraco-Vascolari e Sanità Pubblica - Azienda Ospedale Università Padova	0009-0009-6093-8686
Giulia	Capanna	Fondazione Policlinico Universitario A. Gemelli, IRCCS, UOC Ginecologia Oncologica, Dipartimento per la salute della Donna e del Bambino e della Salute Pubblica, Rome, Italy.	0000-0003-4771-1128
Raffaele	Capoluongo	Azienda Ospedaliera Universitaria Federico II	
Gabriella teresa	Capolupo	Fondazione Policlinico Campus Bio medico di Roma	0000-0002-6009-8180
Filippo Maria	Capomacchia	Ovarian Cancer Center, Candiolo Cancer Institute, FPO-IRCCS, Candiolo, Italy	
Federico	Capone	Università degli Studi di Napoli Federico II	
Alessandro	Capozucco	La Sapienza Roma Sant'Andrea	0009-0002-2711-6284
Federico	Cappellacci	Dipartimento di scienze chirurgiche, Università degli studi di Cagliari	0000-0002-8101-8864
Sonia	Cappelli	Istituto Nazionale Tumori IRCSS Regina Elena Roma. Chirurgia senologica	0000000165879288
Micaela	Cappuccio	Azienda ospedaliera Federico II	
Benedetta	Caproli	Policlinico A.Gemelli	
Vincenzo Francesco	Caputo	Unità operativa di Urologia Ospedale San Carlo di Potenza,	0000-0002-9462-1427
Filippo	Carannante	UOC Chirurgia Colorettale, Fondazione Policlinico Universitario Campus Biomedico di Roma	0000-0003-3618-8431
Ludovico	Carbone	Department of Medicine Surgery and Neuroscience, Unit of General Surgery and Surgical Oncology, University of Siena	0000-0003-2862-6788
Gabriele	Carbone	Ospedale San Raffaele	
Silvia	Cardella	Chirurgia generale 2 Ospedale maggiore della carità di Novara	
Stefano	Cardelli	Dipartimento Medico Chirurgico delle Malattie Digestive, Epatiche ed Endocrino-Metaboliche<br>Chirurgia del Tratto Alimentare ed Urgenze<br>Azienda Ospedaliero-Universitaria di Bologna	0000-0001-8870-5176
Chiara	Caricato	Fondazione Policlinico Universitario Agostino Gemelli	0000-0002-3771-9444
Marco	Carilli	UOSD Urologia Robotica e Mininvasiva - AOU Policlinico Tor Vergata	0000-0002-2416-9762
Silvio	Caringi	Department of Surgery, Università Degli Studi Roma “Tor Vergata”, Via Montpellier 1, 00133 Rome, Italy	0009-0008-6007-8834
Valeria	Carone	Dipartimento di Chirurgia Generale ed Oncologica, A.O.U. Policlinico Umberto I, Università La Sapienza di Roma	0000-0002-4573-3019
Gianmaria	Casoni Pattacini	Ospedale Civile di Baggiovara,Chirurgia Generale,d’urgenza e Nuove Tecnologie	0000-0002-1106-4187

Mario	Casoria	Urologia Federico II	
Gianluca	Cassese	Department of Health Sciences, University of Eastern Piedmont "A. Avogadro", Alessandria, Italy". Se fosse possibile una seconda affiliazione sarebbe "Division of Surgical Oncology, Robotic and HPB Surgery University Hospital of Alessandria, Italy	0000-0002-7127-7440
Andrea	Castaldi	Dipartimento Cardio-Toraco-Vascolare, Unità operativa di Chirurgia Toracica, Università di Pisa	
Antonio	Castaldi	AOU Federico II	0000-0003-3084-7942
Livio	Catozzi	Department of Medicine Surgery and Neuroscience, University of Siena	0000-0003-3441-081X
Margherita	Cattaneo	U.O. Chirurgia Toracica e dei Trapianti di Polmone, Fondazione IRCCS Ca' Granda Ospedale Maggiore Policlinico di Milano	0000-0002-3192-4957
Roberta	Catuzzi	Dipartimento di Chirurgia, UOSD Urologia Robotica e Mininvasiva, Azienda Ospedaliera Policlinico di Tor Vergata	
Ilaria	Ceccarelli	Università di Pisa	
Antonio	Cece	Policlinico Umberto I	
Giuseppe	Celentano	IRCCS Neuromed	0000-0002-3346-8385
Andrea	Celotti	UO chirurgia generale ASST cremona	
Anastasia	Centofanti	Chirurgia toracica vascolare e trapianti d'organo - Paride Stefanini- UOC Chirurgia	
Leonardo	Centonze	1- Department of General Surgery and Transplantation, Niguarda Ca' Granda Hospital, Milan, Italy.<br>2- Clinical and Experimental Medicine PhD Program, University of Modena and Reggio Emilia, Modena, Italy.	0000-0001-9371-7411
Marco angelo	Cereda	Irccs San Gerardo, Monza	0000-0002-8196-8704
Camilla	Certelli	Dipartimento di Scienze della Salute della Donna, del Bambino e di Sanità Pubblica, Fondazione Policlinico Universitario Agostino Gemelli IRCCS, Rome, Italy	0000-0002-8325-6946
Edoardo	Ceruti	Dipartimento di Scienze della Salute della Donna, del Bambino e di Sanità Pubblica, Fondazione Policlinico Universitario Agostino Gemelli IRCCS, Rome, Italy	
Giuliana	Checchini	S.C. Chirurgia Epatica e dei Trapianti di Fegato e Rene, ASST Grande Ospedale Metropolitano Niguarda, Milano, Italia	
Enrico	Checcucci	IRCCS FPO Candiolo Cancer Institute	
Francesco	Chiancone	AORN CARDARELLI UOC UROLOGIA	
Stefano	Chianese	.	
Maria francesca	Chiappetta	Ospedale Sant'Antonio abate Gallarate	
Davide	Chiappori	Chirurgia Generale e dei Trapianti d'organo Ospedale San Camillo Forlanini, Roma	
Francesca	Chicchi	AOU Careggi, Università di Firenze	0009-0002-7148-0227
Federica	Chimenti	IRCCS San Gerardo	0000-0002-6415-8750
Francesco	Cianflone	Department of Urology, Fondazione IRCCS Policlinico San Matteo, Pavia, Italy	0000-0001-7532-9626
Sara	Ciapetti	AOUP Chirurgia plastica e microchirurgia universitaria	
Pierfranco Maria	Cicerchia	Ospedale A. Angelucci Subiaco	0000-0003-0324-7810
Dalia	Cidda	Ospedale San Luigi Gonzaga, Orbassano (Università di Torino)	
Simone	Cilio	U.O.C. Clinica Urologica, AOU San Giovanni di Dio e Ruggi d'Aragona "Scuola Medica Salernitana", Salerno, Italia	0000-0002-8431-9830
Alessandro	Ciolli	UOC Chirurgia Vascolare, Fondazione policlinico universitario Agostino Gemelli	
Cosimo	Ciotta	Ospedale San Pio di Benevento	
Lorenzo	Cirigliano	A.O.U. città della salute e della scienza di Torino - presidio Molinette - Urologia U	
Bruno	Cirillo	Sapienza università di Roma	
Luigi	Cirillo	Department of Neurosciences, Reproductive Sciences and Odontostomatology, University of Naples "Federico II", Naples, Italy	
Cristina	Ciulli	HPB Surgery Unit, Department of General Surgery, IRCCS San Gerardo dei Tintori Foundation, Via Pergolesi<br>33, 20900, Monza, Italy	0000-0003-0911-5114
Francesco	Claps	Oncological Urology - Department of Surgical Oncology - Veneto Institute of Oncology IRCCS	0000-0003-2812-5553
Erminia	Cofano	Department of Orthopaedic and Trauma Surgery, "Magna Graecia" University of Catanzaro,<br>"Renato Dulbecco" University Hospital, 88100 Catanzaro, Italy	
Diego	Coletta	IRCCS REGINA ELENA NATIONAL CANCER INSTITUTE	
Michele	Colloridi	Policlinico Umberto I	
Giovanni	Comacchio	Azienda Ospedale - Università Padova	
Iacopo	Compalati	Aou sant'andrea	
Paolo	Conforri	Università degli studi di Napoli "Federico II", Dipartimento di Neuroscienze	

Luigi Eduardo	Conte	Università degli Studi di Roma "Tor Vergata"	0000-0002-1280-8029
Beatrice	Conti	Policlinico Umberto 1	
Roberto	Contieri	Department of Urology, Istituto Nazionale Tumori di Napoli IRCCS Fondazione "G. Pascale", Napoli, Italy.	
Mariano	Coppola	Urologia Federico II	
Paola	Coppola	Università degli studi della Campania Luigi Vanvitelli unità operativa di urologia	
Diletta	Corallino	Department of General Surgery and Surgical Specialties, Sapienza University of Rome, Viale del Policlinico 155, 00161, Rome, Italy	0000-0002-4937-4332
Giuseppe	Cordaro	Policlinico san Pietro	
Pio	Corleone	Centre Hépatobiliaire, hôpital Paul Brousse	
Claudia	Corrao	.	
Roberta	Corvino	Policlinico Umberto I	
Lorena	Costardi	AO ordine Mauriziano	
Eva	Covello	UO Chirurgia Plastica e Microchirurgia, Università degli Studi di Pisa	
Eleonora	Coviello	Università degli studi di Perugia	
Massimo	Criscione	Women's and Children's Health Department, Complex Unit of Gynecology and Obstetrics, University of Sassari	0000-0002-1627-2444
Giorgio	Crocetti	AOU Careggi	0009-0005-4200-6704
Daniele	Crocetti	Dipartimento di chirurgica, sapienza università di Roma	0000-0001-9950-3553
Rossella	D'Alessio	Chirurgia generale 2 - ASST Spedali Civili di Brescia	0000-0001-5614-7616
Chiara	D'Alterio	Dipartimento di Chirurgia, AOU Policlinico Umberto I, Sapienza Università di Roma	0000-0003-0165-7306
Carlo	D'Alterio	Federico II	
Lorenzo	D'Amico	Specializzando Urologia, Policlinico Gemelli, Roma	
Giuseppe Antonio	D'Amico	Department of Plastic and Reconstructive Surgery, University Hospital of Messina AOU Gaetano Martino, Messina, Italy	0009-0005-1540-2357
Marco	D'Indinosante	Scienze della salute della donna, del bambino e di sanità pubblica, Dipartimento di Ginecologia Oncologica, Fondazione Policlinico A.Gemelli IRCCS	0000-0001-6588-8686
Alberto	Damiano	Dipartimento di Neuroscienze, scienze riproduttive ed odontostomatologiche, Federico II di Napoli	
Federica	Danuzzo	Chirurgia toracica ASST Grande ospedale metropolitano Niguarda	
Licia	Dari	Chirurgia Generale 2U, AOU Città della Salute e della Scienza Torino, Università degli Studi di Torino	0009-0001-6001-4362
Riccardo	De Bernardis	Fondazione Policlinico Universitario Campus Bio Medico di Roma	
Michele	De Capua	Dipartimento di chirurgia, chirurgia generale 2, Spedali Civili di Brescia	0000-0001-6392-3388
Riccardo	De Carlis	ASST Grande Ospedale Metropolitano Niguarda, Milano	0000-0003-3697-1653
Fabio	De Carne	Humanitas	
Alessandra	De Cicco Nardone	Fondazione Policlinico Gemelli IRCCS	0000-0003-4868-3851
Alessia	De Fabiani	Università degli Studi di Milano, Dipartimento di Chirurgia Toracica dell'Istituto Europeo di Oncologia	
Nadia	De Falco	Ospedale Evangelico Betania	
Delia	De Filippo	Azienda Ospedaliera Cardarelli	
Francesco	De Leo	Department of Pediatric Surgery, Fondazione IRCCS Policlinico San Matteo, Pavia, Italy.	0009-0002-4577-6059
Federico	De Leonardis	UOSD di Urologia, Fondazione Policlinico Tor Vergata	0000-0002-8800-9143
Giuseppe Massimiliano	De Luca	Department of Precision and Regenerative Medicine and Jonica Area (Dimepre-J), U.O.C. of General Surgery University " F. Paccione ", University of Bari Aldo Moro, Bari,	0000-0002-9990-5795
Ciro	De Luca	Italy.	
Edoardo	De meis	Specializzazndo di chirurgia generale presso Sapienza università di Roma	0000-0002-7730-2600
Clarissa	De Nardi	Chirurgia Generale 2 - Azienda Ospedaliera di Padova	0000-0002-8177-6358
Valerio	De Peppo	Hepato-bilio-pancreatic and Liver Transplant Unit, Department of Surgery, Sapienza University of Rome, Rome 00161, Italy	0000-0002-1603-0787
Anna	De Rosa	Università Federico ii napoli	
Lorenzo	De Stefano	A.O.U. Salerno	
Noemi	Deanesi	Urologia, Fondazione Policlinico Universitario Campus Biomedico di Roma	
Maria Pia	Del Vecchio	Ginecologia e Ostetricia, AOU Federico II	
Annamaria	Dell' Unto	Ospedale Gaslini	
Giampiero	Della Rosa	Università degli studi di Napoli Federico II	
Antonella	Delvecchio	UOC Chirurgia Epatobiliopancreatica. Ospedale Regionale F. Miulli. Acquaviva delle Fonti (Bari)	0000-0002-7759-4340
Alfredo	Dente	SD chirurgia plastica e Microchirurgia universitaria - AOU Pisana	0009-0005-5387-6377

Francesco	Di Bello	Department of Neuroscience Reproductive Science and Odontostomatology, university of Naples Federico II	0000-0002-8585-3701
Stefano	Di Berardino	UOC Ginecologia Oncologica, Dipartimento di Scienze della Salute della Donna, del Bambino e di Sanità Pubblica, Fondazione Policlinico Universitario A. Gemelli, IRCCS, Rome, Italy	0000-0001-5778-0878
Jasmine	Di Biasi	Fondazione policlinico Gemelli	
Ludovica	Di Cesare	Chirurgia generale e d'urgenza - ospedale Santa Maria Goretti di Latina	
Leonardo	Di Cicco	Department of Medical Surgical Science and Translational Medicine, Sant'Andrea University Hospital, Sapienza University of Rome, Rome, Italy	0009-0005-9652-9930
Roberto	Di Costanzo	Usl toscana sud est	
Camilla	Di Dio	Policlinico Umberto 1	
Giacomo	Di Filippo	Dipartimento di Chirurgia ed Oncologia, UOC Endocrinochirurgia, Azienda Ospedaliera Universitaria Integrata Verona	
Giancarlo	Di Gioia	IRCCS Istituti fisioterapici ospitalieri	
Ivan	Di Giulio	Sapienza università di Roma	
Giovanni	Di Lascio	Urologia - policlinico Umberto I Roma	0000-0003-2925-6934
Dario	Di Lieto	Dipartimento di Neuroscienze, scienze riproduttive ed odontostomatologiche, Federico II di Napoli	
Ernesto	Di Mauro	Department of Neurosciences, Science of Reproduction and Odontostomatology, University of Naples Federico II, 80131 Naples, Italy.	
Giovanna	Di Meo	Dipartimento di Medicina di Precisione e Rigenerativa e Area Jonica, Università degli Studi di Bari	0000-0002-7012-8654
Domenico	Di Nardo	Azienda Ospedaliera Santa Maria di Terni Reparto di Chirurgia Digestiva e d'Urgenza	
Simone	Di Nicola	Chirurgia generale Policlinico Umberto I	
Marco	Di Pangrazio	AOU Modena - Ospedale Civile di Baggiovara - UOC Chirurgia Generale, d'Urgenza e Nuove Tecnologie	
Giovanni Battista	Di Pierro	Dipartimento materno infantile e scienze urologiche,università Sapienza di Roma	0000-0003-2321-0548
Francesco	Di Rocco	Urologia, Policlino Tor Vergata	0009-0007-4808-0520
Davide	Di Seclì	Unità di Chirurgia Plastica, Università degli studi di Siena	
Giada	Di Vincenzo	AOUP (Azienda ospedaliero universitaria Pisana)	
Raquel	Diaz	Department of Surgical and Diagnostic Integrated Sciences-DISC, University of Genova, 16132 Genova, Italy.	
Annalisa	Diddoro	Sapienza di Roma, dip.to scienze chirurgiche	
Gaetano	Digitale Selvaggio	Dipartimento di Neuroscienze, scienze riproduttive ed odontostomatologiche, Federico II di Napoli	
Giuseppe	Diluiso	Università degli Studi di Siena - UOC Chirurgia Plastica, Ricostruttiva ed Estetica	0000-0002-4609-4837
Fabrizio	Dinacci	Department of Neurosciences and Reproductive Sciences and Odontostomatology, University of Naples "Federico II	
Piervito	Dipinto	Sapienza Roma	
Antonio	Dolardo	UOC chirurgia plastica & centro ustioni. Azienda ospedaliera policlinico di Bari.	
Greta	Donisi	Pancreatic Surgery Unit, IRCCS Humanitas Research Hospital, Rozzano, Italy.	
Nassima	Doumit	Policlinico Umberto I	
Francesca	Duro	Chirurgia digestiva, Santa Maria Terni	
Gianmarco	Elia	Azienda Ospedaliera Universitaria Pisana	
Francesco	Esperto	Department of Urology, Campus Biomedico University	0000-0002-0108-1864
Sofia	Esposito	Chirurgia Generale, d'Urgenza e Nuove Tecnologie, ospedale Civile di Baggiovara, AOU Modena	
Giovanni	Esposito	Fondazione Policlino Gemelli IRCCS	0009-0004-1547-4150
Fabio	Esposito	Clinica Lourdes	
Marco	Fabiano	AORN A.Cardarelli	
Ugo	Falagario	1 Department of Molecular Medicine and Surgery, (Solna), Karolinska Institutet, Stockholm, Sweden<br>2 Department of Urology and kidney transplantation, University of Foggia, Foggia, Italy	
Federica	Falaschi	Aorn cardarelli, chirurgia epatobiliare e trapianti di fegato	
Francesco	Falbo	AO San Giovanni Addolorata - Roma	0000-0003-3840-4484
Alfonso	Falcone	Federico II napoli	
Fabrizio	Fanara	Università di Roma Tor vergata	
Lisa	Fantina	Ospedale di Cattinara chirurgia generale	
Chiara	Fantozzi	Università degli Studi di Roma Tor Vergata	0009-0004-7340-7829
Tommaso	Farolfi	Fondazione Policlinico Campus Biomedico	0000-0003-1932-2846
Alessia	Fassari	Università degli Studi di Roma La Sapienza	0000-0001-8534-9823

Matteo	Fatiga	Reparto di Chirurgia maxillo- facciale, Policlinico Umberto 1, Roma	0009-0009-7484-7689
Ludovica	Fattori	AOU Sant'Andrea	
Veronica	Fedele	Varese- fondazione Macchi. Chirurgia d'urgenza e dei trapianti.	0009-0005-8023-4273
Agostino	Fernicola	Azienda Ospedaliera Universitaria Federico II	0000-0002-3122-5531
Piervincenzo	Ferrante Bannera	Dipartimento di patologia umana dell' adulto e del bambino, U.O.C Chirurgia Plastica, Azienda Ospedaliera Universitaria "g. Martino" Messina	0009-0009-2952-0580
Paolo	Ferrari	Policlinico Casilino	
Davide	Ferrari	SS Tumori Ereditari dell'Apparato Digerente, Fondazione IRCCS Istituto Nazionale dei Tumori	0000-0002-7058-5011
Gianpiero	Ferretti	Università federico II	
Lorenzo	Ferri	Dipartimento di Scienze Chirurgiche, UOC Chirurgia Generale, Fondazione Policlinico Universitario A. Gemelli IRCCS	0000-0002-4109-9727
Lucio	Ferriero	Università la sapienza	
Augusto	Ferrini	Fondazione Policlinico Universitario Campus Bio-Medico, Via Alvaro del Portillo 200, 00128, Rome, Italy	0000-0003-0929-3586
Federico	Festa	Chirurgia 1 Ospedale Maria Vittoria ASL Citta' di Torino	0000-0001-7685-588X
Luca	Filipponi	Chirurgia Toracica ASST Sette Laghi Varese	0000-0003-0131-9817
Cristina	Fiorani	Chirurgia Mininvasiva Policlinico Tor Vergata Roma	0000-0002-3188-3366
Ruggiero Antonio	Fiorella	Università di Foggia	
Giorgio	Fiorenza	IRCCS Policlinico Universitario Agostino Gemelli - Università Cattolica de Sacro Cuore	0009-0003-5637-5258
Claudio	Fiorillo	Fondazione Policlinico Universitario A. Gemelli, IRCCS	
Enrico	Fischetti	Azienda Ospedaliero Universitaria Policlinico di Bari	
Rocco Simone	Flammia	IRCCS IFO Regina Elena	
Michele	Fogliata	UO Chirurgia Generale e d'Urgenza Ospedale di Lecco	0009-0000-1035-4003
Alessandro	Fogliati	HPB Surgery Unit, Department of General Surgery, IRCCS San Gerardo dei Tintori Foundation, Via Pergolesi 33, 20900, Monza, Italy	
Caterina	Foppa	Humanitas university; IRCCS humanitas research hospital	0000-0001-9920-0368
Francesca	Forattini	DISCOG, Chirurgia Generale 1^	0009-0004-3066-9159
Laura	Fortuna	Chirurgia Apparato Digerente, AOU Careggi	0000-0003-0624-7445
Marco	Franciullo	Specializzando Urologia Campus bio-medico di Roma	
Domenico	Franco	Fondazione Policlinico Campus Biomedico di Roma	0009-0009-3566-5778
Antonio	Franco	Azienda Ospedaliera Sant'Andrea, Roma, IT	
Pietro	Fransvea	Fondazione Policlinico Universitario A Gemelli IRCCS Roma	0000-0003-4969-3373
Antonio	Franzese	Chirurgia Generale, UOC Chirurgia generale ad indirizzo Bariatrico endocrino metabolico e senologia, aou Federico II Napoli	0000-0001-9060-5406
Camillo	Fulchignoni	UOC ORTOPEDIA E CHIRURGIA DELLA MANO, Fondazione Universitaria Policlinico Agostino Gemelli IRCCS, Roma	0000-0002-1705-7228
Andrea	Fuschi	Dipartimento di scienze e biotecnologie medico chirurgiche - Urologia - Sapienza Università di Roma - Polo Pontino - ICOT di Latina	0000-0002-0194-536X
Giovanni Maria	Fusco	Ospedale S.Maria delle Grazie Pozzuoli ASL Napoli 2 nord	
Giulia	Fusi	Department of Pediatric Surgery, Fondazione IRCCS Policlinico San Matteo, Pavia, Italy	0000-0002-3990-0618
Federica	Galiandro	UOC Chirurgia Addominale, Fondazione Policlinico Universitario "A. Gemelli" IRCCS Roma	0000-0001-5041-4867
Filippo Tommaso	Gallina	IRCCS Istituto Nazionale Tumori Regina Elena	0000-0001-5579-0837
Giacomo	Gallo	UOC Urologia, AOU Sant'andrea, Università La Sapienza di Roma	
Maria gloria	Gallotti	Libero professionista	
Antonio	Gargiulo	A.O.U. Federico II di Napoli	0009-0009-3527-0119
Eleonora	Garofalo	Ospedale San Camillo forlanini	
Camilla	Gasparoli	Chirurgia generale e dei trapianti, ospedale San Camillo	
Carlo	Gazia	Ospedale San Pietro Fatebenefratelli	0000-0002-3543-4170
Enza	Gelormini	Policlinico Umberto I; Università di Roma La Sapienza	0009-0007-2403-4706
Alessandro	Gentilucci	ASL Viterbo Ospedale Santa Rosa UOC Urologia	
Antonella	Geraci	Aslnapoli3sud	
Omar	Ghazouani	Chirurgia generale, Ospedale Santa Corona, Pietra Ligure	0000-0003-1075-0342
Gaetano	Giampaglia	Asl Napoli 1 Centro - Ospedale del Mare	
Simone	Gianazza	UOC Chirurgia Generale, Ospedale Galmarini Tradate, ASST Settelaghi	0000-0001-9270-1709
Alessandro	Giani	ASST Grande Ospedale Metropolitano Niguarda, Division of Minimally-invasive Surgical Oncology, Piazza Ospedale Maggiore, 3 20162, Milan, Italy	0000-0003-0718-2321
Rachele Anna	Giannella	.	
Luca	Giordano	Ospedale San Pietro Fatebenefratelli, Roma	0009-0003-1842-5680
Alessandro	Giordano	Università degli studi Federico II	
Francesco	Giovanardi	Uosd chirurgia d'urgenza- PTV	0000-0002-8395-2722

Carlo	Giove	Policlinico di Bari	
Gennaro	Giovine	U.O.C. Chirurgia oncologica mininvasiva, A.O.U. Federico II di Napoli	0000-0002-1121-8406
Veronica Maria	Giudici	Chirurgia Toracica, IRCCS Humanitas Research Hospital	
Mario	Giuffrida	Department of General Surgery, Piacenza General Hospital, Piacenza, Italy.	0000-0001-8335-3941
Michele	Giugliano		
Carlo	Giulioni	Casa di cura Villa Igea	
Giancarlo	Giurazza	Fondazione Policlinico Universitario Campus Bio-Medico	0000-0003-4385-6813
Simone	Giusti	Fondazione Policlinico Universitario Agostino Gemelli IRCCS	0009-0007-8280-5222
Luca Matteo	Gobbi	Sapienza Università di Roma	
Marta	Goglia	Medical and Surgical sciences and Translational Medicine Department, Sapienza University of Rome	0000-0002-3763-8847
Luca	Gordini	Chirurgia Generale e Robotica	
Lorenzo	Gozzini	IRCCS San Raffaele scientific Institute	0000-0001-6605-4683
Vlad Marian	Gradinaru	Non	
Grazia	Granata	Università degli studi di Roma Tor Vergata	
Roberta	Granata	Dipartimento di emergenze medico-chirurgiche, U.O.C. Chirurgia Generale III, A.O.U. Policlinico G-Rodolico-S.Marco Catania	0000-0003-1857-0805
Giuliano	Granata	Università degli studi di Napoli Federico II	
Arrigo	Grasso	Università di Pisa	
Damiano	Graziani	Specializzando Urologia Sapienza - Polo Pontino	
Antonio	Greco	Policlinico Roma Tor Vergata	
Elena	Guadagni	Università di Pisa	
Giorgia	Gualtieri	Chirurgia Colorettale Istituto clinico Humanitas	0000-0002-9591-1856
Annalisa	Guarracino	Chirurgia Generale, AOU Santa Maria delle Scotte	
Catia	Gugliucciello	Università' La Sapienza	
Alessio	Guidotti	La Sapienza Università di Roma - Scuola di Specializzazione in Urologia - Sede Sant'Andrea (Roma)	
Giacomo	Guidi	Fondazione Policlinico Universitario Agostino Gemelli IRCCS - Università Cattolica del Sacro Cuore	0009-0002-2162-9863
Sara	Guzzetti	Azienda ospedaliera Santi Carlo e Paolo, presidio San Carlo Borromeo	0000-0002-7936-4477
Redan	Hassan	Azienda Ospedaliera San Camillo Forlanini	
Ilda	Hoxhaj	Department of Medical and Surgical Sciences, Fondazione Policlinico Universitario Agostino Gemelli, Istituto di Ricovero e Cura a Carattere Scientifico (IRCCS), 00168 Rome, Italy.	0000-0001-7086-2456
Alessandro	Iacomino	Chirurgia 2 - Ospedale Regionale Ca Foncello di Treviso	
Salvatore	Iaconis	Università degli studi di Napoli Federico II	
Immacolata	Iannone	Dipartimento di Chirurgia AOU Policlinico Umberto I - Roma	0000-0001-9564-7306
Eva	Iannuzzi	Policlinico Umberto primo	
Fausto	Illiano	Federico Secondo	
Silvestro	Imperatore	Endoscopia	
Sara	Ingallinella	Hepatobiliary Surgery Division, IRCCS San Raffaele Hospital, Via Olgettina 60, 20132, Milan, Italy.	
Simona	Ingrassano	1 Urology Clinic, Department of Surgical Sciences, City of Health and Sciences at Molinette University Hospital, University of Turin, Turin, Italy.	0000-0001-8210-9752
Chiara	Innocenzi	2 Urology Section, University of Catania, Catania, Italy.	0009-0001-6997-9099
Maria	Iorio	Uoc ginecologia ospedale San Giuseppe	
Angela	Iovine	UOC chirurgia toracica, AOU Vanvitelli	
Carlotta	La Raja	Coloproctology and Pelvic Floor Surgery Unit, IRCCS Policlinico San Donato, San Donato Milanese, Metropolitan City of Milan, Italy	0000-0003-4338-596X
Andrea	La Rosa	Department of Surgical, Medical and Molecular Pathology and Critical Care Medicine, University of Pisa, 56124 Pisa, Italy.	0009-0009-8489-7057
Guido	Lancellotti	Ospedale A Gemelli	
Nunzio Alberto	Langella	AORN Cardarelli	
Maria	Lanzone	Chirurgia d'urgenza- Policlinico Umberto I, Roma	
Giovanni Guglielmo	Laracca	AOU Sant'Andrea, Roma	0000-0002-4508-2180
Ilario	Lattina	Aurelia Hospital chirurgia generale	
Sara	Lauricella	Fondazione IRCCS Istituto Nazionale dei Tumori, Milano	0000-0002-3295-6264
Antonio	Laurino	Fondazione Policlinico Gemelli Roma	0000-0002-3344-4279
Giulia	Lauteri	San Carlo di Nancy Roma, La sapienza	0009-0003-9349-335X
Federico	Lavagno	Università degli Studi di Torino	
Arturo	Lecce	U.O.C urologia ospedale del mare	
Dario	Leto	Università degli Studi di Messina, U. O. C. di chirurgia plastica	0009-0001-8802-9048

Francesco Saverio	Li Causi	Department of Medical Surgical Science and Translational Medicine, Sant'Andrea Hospital, Sapienza University of Rome, 00198 Rome, Italy	0009-0006-0592-7616
Gloria	Licchettq	Università degli studi di milano	
Luigi	Lione	Azienda ospedaliera universitaria di Padova	
Mattia	Lo Re	AOU CAREGGI, università di firenze	
Sara	Lo Torto	Fondazione IRCCS San Gerardo dei Tintori di Monza	0009-0009-6581-1692
Eleonora	Locci	Chirurgia Bariatrica, ARNAS Brotzu Cagliari	0000-0001-5309-2138
Alessandro	Lombardi	Università degli studi di Napoli "Federico II"	
Maria Chiara	Longo	Università Vita - Salute San Raffaele	0009-0006-1251-326X
Giustina	Lopopolo	Policlinico Universitario Agostino Gemelli	
Eleonora	Lori	Dipartimento di Chirurgia, Sapienza Università di Roma	0000-0003-1943-4952
Francesco Saverio	Loria	Policlinico Umberto I,Università di Roma "La Sapienza"	
Luigi	Losco	Dipartimento di Medicina, Chirurgia e Odontoiatria. Università degli studi di Salerno	0000-0001-9105-1839
Pasquale	Losurdo	SC Chirurgia Generale, Azienda Sanitaria Giuliano-Isontina, Ospedale di Monfalcone	0000-0002-7230-2046
Andrea	Lovece	IRCCS Policlinico San Donato	0000-0002-5176-7303
Matteo	Loverro	Fondazione Policlinico Universitario A. Gemelli, IRCCS, Roma, Italia	0000-0002-9740-3169
Antonio	Luberto	IRCCS Humanitas Research Hospital, Division of Colon & Rectal Surgery, Rozzano, Milan, Italy.	0000-0002-1202-9447
Alessio	Lucarini	Surgical and Medical Department of Translational Medicine, Sant'Andrea Hospital, Sapienza University of Rome, Via di Grottarossa, 1035, 00189 Rome, Italy	0000-0002-9644-740X
Sara	Lucchese	Ospedale SS Maria dello Splendore, Giulianova, ASL 4 Teramo	0000-0002-1807-4513
Fabiola	Lucchino	Ospedale San Camillo Roma	
Andrea-Pierre	Luzzi	Asst Santi Paolo e Carlo	0000-0003-4218-3848)
Fabio	Machiella	Neuroscienze Federico II	
Isabella	Madaffari	UOC Chirurgia generale. Azienda ospedaliera universitaria Sant'Andrea	
Antonio	Madonna	Dipartimento di Neuroscienze, scienze riproduttive ed odontostomatologiche, Federico II di Napoli	
Francesco	Maffia	Maxillofacial Surgery Unit, Department of Neurosciences, Reproductive and Odontostomatological Sciences, University of Naples "Federico II", 80138 Naples, Italy	0000-0003-1293-2107
Sara	Magaletti	Università degli studi di Roma "Sapienza" - Policlinico Umberto I	0009-0005-3699-7548
Riccardo	Magarini	Chirurgia oncologica e mininvasiva ospedale Niguarda	0000-0002-8125-5018
Federico	Maggi	Ospedale San Carlo di Nancy, Roma, UOC Chirurgia Generale	0009-0002-4103-3979
Martina	Maggi	Unit of Urology, Department of Health Science, ASST Santi Paolo and Carlo, Milan, Italy	
Marco	Magliocchetti	Dipartimento di Neuroscienze, scienze riproduttive ed odontostomatologiche, Federico II di Napoli	
Cecilia	Maina	Ospedale San Raffaele	0000-0002-8754-1007
Riccardo	Mainenti	Azienda ospedaliera universitaria senese santa Maria le scotte	
Enrico	Maisto	U.O.C di Urologia, Ospedale Antonio Cardarelli	
Sofia	Maliziola	Dipartimento di Urologia, Policlinico di Tor Vergata Roma	
Valerio	Mallia	Department of Biomedical Sciences, Humanitas University, via Rita Levi Montalcini 4, 20072 Pieve Emanuele, Milan, Italy	
Girolamo	Maltese	Fondazione Policlinico Campus Bio-Medico, Ortopedia e Traumatologia	
Michele	Manara	Dipartimento Chirurgia Generale, U.O. Chirurgia Generale, I.R.C.C.S. Galeazzi - Sant'Ambrogio	0000-0002-4924-1919
Celeste	Manfredi	Urology Unit, Department of Woman, Child and General and Specialized Surgery, University of Campania "Luigi Vanvitelli", Naples, Italy	0000-0002-9706-8516
Beatrice	Manfredini	Minimally Invasive and Robotic Thoracic Surgery, Surgical, Medical, Molecular, and Critical Care Pathology Department, University of Pisa, 56126 Pisa, Italy	0009-0001-9932-5117
Michele	Manigrasso	DAI di Chirurgia Generale, dei Trapianti e Gastroenterologia, U.O.C di Chirurgia Endoscopica, Azienda Ospedaliero-Universitaria Federico II di Napoli	0000-0001-8204-7942
Mattia	Manitto	U.O.C. Chirurgia toracica, Policlinico IRCCS, Genova	0000-0001-5990-0933
Monica	Mansi	Policlinico Umberto I- "Sapienza" università di Roma	0000-0003-2694-1063
Guglielmo	Mantica	Department of Surgical and Diagnostic Integrated Sciences, University of Genova, Genova, Italy	0000-0003-4361-4523
Federica Rita	Marano	Humanitas University	
Michele	Marchioni	University degli Studi "G. d'Annunzio" di Chieti e Pescara	0000-0002-1702-4127
Maria Cristina	Marconi	ASST Mantova Ospedale Carlo Poma	
Germano	Mariano	Ospedale San Camillo - Forlanini POIT	

Davide	Marino	Chirurgia oncologica e d'urgenza, Policlinico Casilino - Università degli studi di Roma Tor Vergata	0000-0001-8438-4774
Annarita	Marino	Uoc chirurgia pediatrica, azienda ospedaliera di Cosenza	0000-0001-6989-7037
Cecilia	Marino	Università degli studi di Milano	
Martina	Marrelli	Campus Biomedico	0000-0002-6268-0502
Luca	Marsero	Ospedale di Verduno aslcn2	
Arianna	Martinelli	Fondazione Policlinico Universitario Campus BioMedico di Roma	
Raffaele	Martino	Ospedale del Mare	
Alessia	Martoccia	Urologia, ospedale NOC, asl Roma 6	
Gianluca	Mascianà	UOC Chirurgia Colo-rettale. Fondazione Policlinico Universitario Campus Bio Medico di Roma	0000-0001-5853-9023
Mosè	Maspero	Ospedale Niguarda, chirurgia 2	0000-0003-3865-3157
Riccardo	Masserano	Asst santi Paolo Carlo, Milano<br>Ospedale San Paolo	
Fanny	Massimi	Chirurgia Generale, Azienda Ospedaliera-Universitaria Sant'Andrea, Università La Sapienza di Roma	0009-0003-3560-7064
Francesco	Mastrangelo	Università Federico II DI NAPOLI	
Marco	Materazzo	Dipartimento di Scienze Chirurgiche, Università degli studi di Roma Tor Vergata	0000-0002-3599-061X
Matteo	Santoliquido	IRCCS Ospedale Policlinico San Martino - Genova	0000-0002-8533-9040
Giovanni	Mattioni	School of Thoracic Surgery, University of Milan, Milan, Italy	0000-0003-4136-8293
Gennaro	Mazzarella	Division of General and Hepatopancreatobiliary Surgery, IRCSS Regina Elena National Cancer Institute of Rome, Rome, Italy	0000-0002-1206-9051
Alessandro	Mazzola	Fondazione Policlinico Universitario Campus Bio-Medico, Via Alvaro del Portillo, 200 - 00128 Roma, Italy	0000-0002-0179-109X
Rossella	Melcarne	Dipartimento di Medicina Traslazionale e di Precisione, Università di Roma Sapienza	
Piero Giuseppe	Meliante	Ospedale "V. Monaldi", Azienda Ospedaliera dei Colli, Napoli; "Sapienza" Università di Roma, dipartimento NESMOS, Ospedale "Sant' Andrea", Roma	0000-0002-8762-3731
Federica	Mellone	Ospedale policlinico San Martino	
Simona	Meneghini	Dipartimento di chirurgia generale, unità di chirurgia d'urgenza e trauma, policlinico Umberto I, Roma	0000-0002-0287-8517
Marta	Meneguzzi	Chirurgia Toracica Azienda Ospedaliera Sant'Andrea	
Alessio	Merendoni	Università degli studi di Roma Tor Vergata	0000-0002-6348-8870
Fabiana	Messa	Azienda ospedaliero universitaria Sant Andrea	Si
valentina	miacci	Fondazione policlinico universitario campus biomedico di Roma	0000-0001-7158-9068
Rosario	Micali	Policlinico universitari Tor Vergata	
Francesca	Miceli	Dipartimento Chirurgia Generale e specialistica, UOC chirurgia vascolare, azienda ospedaliera universitaria Policlinico Umberto I, Università Sapienza.	
Nicola	Migliaccio	Cardarelli	
Michele	Migliorini	Università degli Studi di Pavia	0000-0003-1726-2341
Nicolò	Mininni	Università degli Studi di Bari "Aldo Moro" - AOU Policlinico di Bari	
Fabio	Mirto	U.O. Urologia Robotica ed Endoscopica, Pineta Grande Hospital, Via Domitiana Km 30/00, Castelvolturno (CE)	0009-0000-3553-000X
Salvatore	Mola	Federico II	
Mattia	Molteni	Department of Robotic and Emergency Surgery, Alessandro Manzoni Hospital, ASST Lecco, Lecco, Italy	0000-0003-1471-439X
Beatrice	Molteni	Chirurgia generale ASST-Franciacorta	0000-0002-1819-4750
Francesca	Montanaro	Dipartimento di Urologia AOUI Verona	
Paolo	Montanelli	UOC Chirurgia Epatobiliare e Trapianti d'Organo, Policlinico San Martino	
Nicola	Montemurro	Dipartimento di Neuroscienze, UO Neurochirurgia, Azienda Ospedaliero Universitaria Pisana, Pisa, Italy	0000-0002-3686-8907
Mauro	Montuori	Dipartimento di Chirurgia Generale, Policlinico San Pietro	0000-0002-7968-2109
Michele	Morelli	Ospedale Maggiore AUSL Bologna	0000-0002-2680-1776
Vincenzo	Morgera	Federico 2	
Martina	Moriconi	Department of Maternal-Infant and Urological Sciences, "Sapienza" Rome University, Policlinico Umberto I Hospital, Rome, Italy	0009-0003-7008-5994
Ambra	Moriconi	Fondazione Policlinico Universitario A. Gemelli, IRCCS, UOC Ginecologia Oncologica, Dipartimento per la salute della Donna e del Bambino e della Salute Pubblica, Roma, Italy.	
Simone	Morra	Azienda Ospedaliera Universitaria Policlinico Federico II di Napoli	0000-0001-5340-8569
Giovanni	Moschetta	CHIRURGIA GENERALE E D'URGENZA SUBIACO	
Emilia	Mottola	UOC chirurgia toracica San Giuseppe moscati Avellino	
Silvia	Muraca	Policlinico Agostino Gemelli	
Michele	Musone	Michele-musone@libero.it	
Baudolino	Mussa	Dipartimento di scienze chirurgiche	0000-0002-0550-1002

Edoardo Maria	Muttillo	Sapienza Università di Roma	
Antonio	Nacchia	Azienda Ospedaliero-Universitaria S.Andrea di Roma	0000-0002-3985-1991
Dania	Nachira	Department of General Thoracic Surgery, Fondazione Policlinico Universitario "A.Gemelli" IRCCS, Università Cattolica del Sacro Cuore, Rome, Italy	
Jacopo	Nanni	Università degli Studi “Magna Graecia” di Catanzaro	
Luigi	Napolitano	Azienda sanitaria locale Salerno	
Niccolò	Nardi	Policlinico Campus Bio-Medico, Unità operativa Ortopedia e Traumatologia	
Giulio Maria	Natali	Policlinico Casilino Università di Tor Vergata	0009-0000-3612-2120
Giovanni Francesco	Niccolini	Policlinico Umberto I Otorinolaringoiatria dipartimento testa collo	0000-0002-1973-3031
Alice	Nigro	Policlinico Casilino	
Riccardo	Oliva	Gynecologic Oncology Unit, Department of Woman and Child Health and Public Health, Fondazione Policlinico Universitario A. Gemelli IRCCS, Università Cattolica Del Sacro Cuore, Roma, Italy; IRCAD, Research Institute Against Digestive Cancer, Strasbourg, France	0000-0001-5251-8133
Alba	Oliva	Ospedale Evangelico Internazionale	
Greta	Olivari	Chirurgia Generale, ospedale San Raffaele di Milano	
Alberto	Olivero	Urologia, ASST Grande Ospedale Metropolitano Niguarda	0000-0001-8703-9597
Mauro	Oltolina	Università degli studi di Milano	0000-0002-5659-9572
Paolo	Ossola	Chirurgia Generale 2U, Città della salute e della scienza, Torino	0000-0003-2944-3916
Luca	Ottaviani	ASST Cremona	0000-0002-4846-1354
Martina	Ottaviani	Unita di ginecologia	
Luca	Pace	Gynecology and Obstetrics Unit, Department of Surgical Sciences, Umberto I Hospital, University of Turin, Turin, Italy.	0000-0003-3076-3909
Marco	Paciotti	Department of Urology, IRCCS Humanitas Research Hospital Rozzano, Milan, Italy;<br>Department of Biomedical Sciences, Humanitas University, Milan, Italy	0000-0002-8456-6495
Giovanni	Pagano	Azienda ospedaliera universitaria Federico 2	
Maria Rosaria	Pagano	Policlinico Federico II	
Giulia	Pagliarini	Dipartimento di scienze cardio-toraco-vascolari e sanità pubblica, chirurgia toracica, università di Padova	0009-0009-9006-4684
Daniele	Paglione	Chirurgia generale "falcone", dipartimento polispecialistico, azienda ospedaliera di Cosenza	0000-0003-3922-6552
Giulio	Pagnani	Dipartimento di Scienze Odontostomatologiche e Maxillo-Facciali, UOC di Chirurgia Maxillo-Facciale, Azienda Ospedaliero Universitaria Policlinico Umberto I, Università La Sapienza, Roma	
Erika	Palagonia	Niguarda	
Caterina	Palestini	Chirurgia Generale - Policlinico A. Gemelli	
Angela	Palma	Department of Maxillo-Facial Surgery, Azienda Ospedaliero-Universitaria Policlinico Umberto I, Viale del Policlinico 155, 00161, Rome, Italy	
Luca	Palmieri	Università Cattolica del Sacro Cuore, Policlinico Universitario Agostino Gemelli IRCCS, Dipartimento di Ostetricia e Ginecologia	0000-0002-9704-3300
Veronica	Palombi	Policlinico Militare di Roma	0000-0002-9246-5459
Marco	Palucci	S.C.D.U. Chirurgia robotica, oncologica ed epatobiliopancreatica, Azienda Ospedaliero-Universitaria SS. Antonio e Biagio e Cesare Arrigo Alessandria	0009-0009-8393-6667
Giovanni	Panico	Fondazione Policlinico Universitario A. Gemelli IRCCS, UOC Ginecologia Oncologica, Dipartimento di Scienze della Salute della Donna e del Bambino e di Sanità Pubblica, Roma, Italia, 00168	
Giuseppe Francesco	Papalia	Research Unit of Orthopaedic and Trauma Surgery, Department of Medicine and Surgery, Università Campus Bio-Medico Di Roma, Via Alvaro del Portillo 21, 00128 Rome, Italy	0000-0002-4140-738X
Salvatore	Papi	Urologia Vanvitelli	
Marco	Paratore	Academic Unit of Obstetrics and Gynecology, IRCCS Ospedale Policlinico San Martino, 16132 Genova, Italy	
Sara	Parini	SCDU Chirurgia Toracica, Ospedale Maggiore della Carità di Novara	0000-0002-6021-6247
Francesco Rosario	Parisi	Research Unit of Orthopaedic and Trauma Surgery, Department of Medicine and Surgery, Università Campus Bio-Medico Di Roma, Via Alvaro del Portillo 21, 00128, Rome, Italy.	0000-0003-2637-3668
Giuseppe	Parlongo	Chirurgia Generale e d’Urgenza GOM BMM RC	
Alessandro	Pasculli	Dipartimento di Medicina di Precisione e Rigenerativa e Area Jonica. Università degli Studi di Bari "A. Moro". UOC Chirurgia Generale Universitaria "F. Paccione	
Rocco	Pasqua	Sapienza, Università di Roma	0000-0003-3698-7348
Francesco	Passaro	Pascale	

Erika	Passone	Ospedale Maggiore della Carità Novara, chirurgia toracica	
Francesco	Pata	Dipartimento di Farmacia e di Scienze della Salute e della Nutrizione, Università della Calabria, Rende (CS)	0000-0003-2634-1199
Alexandro	Patirelis	Unit of Thoracic Surgery, Department of Surgical Sciences, Tor Vergata University Polyclinic, Viale Oxford, 81, 00133 Rome, Italy	0009-0009-7409-8470
David	Pavoncello	Policlinico di Tor Vergata	
Matteo	Pavone	Dipartimento di Scienze per la salute della Donna e del Bambino e di Sanità Pubblica, UOC Ginecologia Oncologica,Fondazione Policlinico Universitario A. Gemelli, IRCCS, Rome, Italy	0000-0002-9791-9464
Giovanna	Pavone	Dipartimento di Scienze Mediche e Chirrigiche, SC Chirirgia Generale Universitaria, Università di Foggia, Policlinico Riuniti Foggia	0000-0003-2523-4914
Simone	Pavone	Humanitas University	0009-0005-6586-7179
Giulia	Pellecchia	Dipartimento di Medicina, Università degli Studi di Udine	0000-0002-0644-0301
Antony	Pellegrino	IRCCS Ospedale San Raffaele	0000-0002-0898-1953
Marco	Pellicciaro	Breast Unit PTV, Dipartimento di scienze chirurgiche Università degli studi di Roma Tor Vergata	0000-0001-9557-2850
Enrica	Pellizzer	Dipartimento di scienze cardio-toraco-vascolari e sanità pubblica, Chirurgia Toracica, Università degli Studi di Padova, Azienda Ospedaliera di Padova	0009-0000-6612-6480
Beatrice	Pelloni	Università di Modena e Reggio emilia	0009-0009-0955-3959
Francesco	Pepillo	Federico II Napoli	
Bruno	Perotti	UOC chirurgia generale - Ospedale Versilia - AUSL Toscana Nord-Ovest	0000-0003-0163-3582
Giampaolo	Perri	Chirurgia epatobiliopancreatica e dei trapianti di fegato dell'Azienda Ospedale Università di Padova, Dipartimento di scienze chirurgiche, oncologiche e gastroenterologiche (DISCOG) dell' Università di Padova	0000-0002-2072-7114
Emanuele	Perrone	Fondazione Policlinico Universitario A. Gemelli, IRCCS, UOC Ginecologia Oncologica, Dipartimento per la salute della Donna e del Bambino e della Salute Pubblica, Roma, Italy.	0000-0002-0938-2493
Giulio	Perrone	Chirurgia endocrina e metabolica policlinico A.Gemelli	
Francesco	Persico	A.O.R.N. A. CARDARELLI NAPOLI, UOSC UROLOGIA	
Marianna	Peruffo	Division of Vascular Surgery, Department of Experimental and Clinical Medicine, University of Florence	0009-0000-6327-8139
Lorenzo	Petagna	UO Chirurgia Epatica e del Trapianto di Fegato - AOU Pisana	0000-0003-0467-550X
Giusy	Petti	Chirurgia Generale Università degli studi di Napoli Federico II	
Mario	Pezzella	Dipartimento di scienze cardio-toraco-vascolari e sanità pubblica, Università degli studi di Padova	0009-0000-1477-5331
Arcangelo	Picciariello	Department of Experimental Medicine, University of Salento, Lecce, Italy	0000-0002-6361-1111
Stefania Angela	Piccioni	Department of Medicine Surgery and Neuroscience, University of Siena	
Giorgia	Piccioni	UOC Chirurgica Toracica, AOU S Andrea, Università di Roma La Sapienza	
Salvatore	Piemonte	AOU Federico II	
Diletta Maria	Pierazzi	Breast Unit Arezzo, Azienda Toscana Sud Est	
Jacopo	Pieri	Università di Bologna, Policlinico Sant'Orsola Malpighi, UO Urologia Brunocilla	
Michele	Pierro	Dipartimento di Scienze della salute della donna, del bambino e di sanità pubblica,UOC Ginecologia, Università Cattolica del sacro cuore di Roma	0009-0003-4697-5305
Domenica	Pignatelli	AOU Federico II	0000-0003-0755-7269
Enrico	Pinotti	Chirurgia Generale ed Oncologica Policlinico San Pietro, Ponte San Pietro Bergamo	0000-0003-3063-4170
Carla	Piras	Università degli studi di Cagliari, dipendente asl Sulcis, CTO di Iglesias, Chirurgia Generale	0000-0002-7706-9159
Fulvia	Pirrelli	Fondazione universitaria policlinico gemelli	
Ludovica	Pitoni	Breast surgery, IRCCS Ospedale San Raffaele	0000-0001-8462-8695
Matteo	Pittacolo	Azienda Ospedale Universita Padova, Chirurgia Generale 1	0000-0002-3938-289X
Sara	Poggi	Plastic Surgery Unit, Department of Medicine, Surgery and Neuroscience, University of Siena, 53100 Siena, Ital	
Gaetano	Poillucci	UOC Chirurgia Generale, Mininvasiva e Robotica - Ospedale San Matteo degli Infermi - Spoleto - USL Umbria 2	0000-0002-3961-1904
Alice	Poli	Ginecologia e Ostetricia ospedale Santa Maria della Misericordia di Udine	
Mirco	Pozzi	Plastic Surgery and Microsurgery Unit, Department of Translational Research and New Technologies in Medicine and Surgery, University of Pisa, 56126 Pisa, Italy.	

Francesco	Prata	Department of Urology, Fondazione Policlinico Universitario Campus Bio-Medico, Via Alvaro del Portillo, 200, Roma, 00128, Italy	0000-0003-3084-6210
Marianna	Praticò	A.O. San Giovanni Addolorata	0009-0000-4862-6027
Sara	Pregnolato	DMED, ginecologia e ostetricia, università degli studi di Udine	
Fabrizio	Presicce	UOC Urologia - Ospedale San Filippo Neri, ASL RM 1, Roma, Italia	
Chiara	Pretini	Chirurgia Generale, Campus biomedico di Roma	
Priscilla	Procopio	Fondazione Policlinico Universitario Agostino Gemelli IRCCS	0000-0002-1916-8335
Luca	Properzi	Università di Perugia	
Giacomo	Puca	Specializzando	
Ilaria	Puccica	IFO - Regina Elena	0009000525089552
Guenda	Pulcina	Azienda Ospedaliera Romagna	
Andrea	Quazzico	Policlinico Riuniti di Foggia	0009-0001-8708-6681
Giuseppe	Quero	UOC Chirurgia Digestiva, Fondazione Policlinico Universitario Agostino Gemelli IRCCS di Roma	
Lorenzo	Ramaci	No	
Michele	Randazzo	UOC UROLOGIA POLICLINICO G.rodolico di Catania	
Alessandro	Ranieri	AOU Federico II	
Maria Eliana	Rapicano	Urologia - AOU Federico II	
Elvira	Rapisarda	Policlinico Universitario Agostino Gemelli	
Eleonora	Razeto	Materno infantile e scienze urologiche università La Sapienza di Roma, Umberto I	
Pasquale	Reccia	AORN dei Colli, Ospedale Monaldi di Napoli	
Andrea	Renne	Dipartimento di chirurgia generale, Chirurgia generale ad indirizzo emergenziale, Università degli studi di Napoli Federico II	
Onofrio Antonio	Rera	Dipartimento di scienze e biotecnologie medico chirurgiche - UOC di Urologia - Università degli studi La Sapienza di Roma - Polo Pontino - ICOT di Latina	
Stefano	Restaino	Azienda Sanitaria Universitaria Friuli Centrale	0000-0002-7848-0329
Filippo	Riccardo	Ospedale santa Maria delle grazie	
Camilla	Riccetti	Policlinico Agostino Gemelli IRCSS	
Marco	Ricci	UOC Urologia, Policlinico Universitario Campus Bio-medico di Roma	0009-0002-2508-2391
Luigi	Riello	Azienda ospedaliera Federico secondo ginecologia e ostetricia	
Valerio	Rinaldi	Sapienza Università di Roma	0009-0005-1778-9399
Sara	Riolo	La Sapienza Università di Roma	
Lorenzo	Ripamonti	Fondazione IRCCS San Gerardo dei Tintori	0000-0002-3762-4285
Martina	Robustelli	UOC Chirurgica Toracica, Università degli studi della Campania Luigi Vanvitelli	
Maura	Rocchetti	Policlinico Umberto I, università di Roma la Sapienza	0000-0001-6783-1907
Angela	Romano	IRCCS sant'orsola Malpighi Bologna	0000-0002-5828-710X
Francesco	Romano	Ospedale del Mare	
Giuseppe	Romeo	Ospedale AORN "A.Cardarelli" - Napoli	
Costanza	Romeo	Dipartimento Scienze della salute della donna, del bambino e di sanità pubblica, Fondazione Policlinico Agostino Gemelli, Università Cattolica del Sacro Cuore	
Davide	Rosati	Azienda ospedaliera universitaria, policlinico Umberto I	
Lorenzo	Rosi	Ospedale San Raffaele	
Francesco	Rossi	Dipartimento di Urologia, Fondazione Policlinico Universitario Agostino Gemelli IRCCS	0009-0003-5141-7527
Daniela	Rossiello	Chirurgia generale Asp Cosenza	0000-0002-1747-0370
Andrea	Rubinacci	Università degli studi di Napoli Federico II	
Marina Serena	Rubino	Università degli studi di Salerno	0009-0000-7861-1352
Antonio	Ruffo	Dipartimento di Medicina e di Scienze della Salute "Vincenzo Tiberio", Cattedra di Urologia<br>Università degli Studi del Molise (CB)	
Alessandro Ferdinando	Ruffolo	CHU Lille, Service de chirurgie gynécologique, F-59000 Lille, France	0000-0002-9983-523X
Pierluigi	Russo	Department of Urology, IRCCS A. Gemelli University Polyclinic Foundation, Department of Life Science, Health, and Health Professions, Unilink University	0000-0002-7789-9486
Silvio Andrea	Russo	Università Cattolica del Sacro cuore, policlinico Agostino Gemelli	0009-0000-9060-1501
Salvatore	Russo	Ospedale di Erba	0009-0004-1099-9249
Filippo Antonino Maria	Saccà	Azienda ospedaliera integrata di Verona Borgo Trento	
Luca	Sacco	UOC Chirurgia Generale e d'Urgenza Ospedale "Santo Spirito" Pescara	

Gabriele	Saccone	Dipartimento di Neuroscienze, Scienze Riproduttive ed Odontostomatologiche, Scuola di Medicina, Università di Napoli Federico II	
Giacomo	Salina	Università La Sapienza	
Fabiana	Salvati	Ginecologia Oncologica	
Giovanni	Salzano	Dipartimento di Neuroscienze e Scienze Riproduttive e Odontostomatologiche. UOC di Chirurgia Maxillo Facciale . Aou Federico II Napoli	
Viola	Sambataro	Dipartimento di scienze cardio-toraco-vascolari, Chirurgia Toracica, Azienda Ospedaliera Università di Padova	
Federico	Sangiuolo	Chirurgia Oncologica Ospedale Ss. Antonio e Biagio e Cesare Arrigo	0009-0006-4724-5138
Catello	Sansone	Università degli Studi di Napoli Federico II	
Maria Sara	Santicchia	Università degli Studi di Roma Tor Vergata	0009-0002-1984-2913
Marco	Santoboni	Università La Sapienza	
Pietro	Santocchi	Chirurgia Oncologica e d'Urgenza, Policlinico Casilino, Roma	0009-0003-1321-2491
Lorenzo	Santodirocco	Sapienza Università di Roma	
Gianmarco	Saponaro	UOC Chirurgia Maxillo Facciale Fondazione Policlinico Gemelli IRCSS	
Marco	Sarcinella	Università degli studi di Napoli Federico II	
Paolina	Saullo	Dipartimento di Chirurgia, Azienda Ospedaliera Policlinico Umberto I, Sapienza Università di Roma	0000-0003-3694-1761
Cosimo	Saviello	UOC Chirurgia Generale e d'Urgenza, PO Santa Maria della Speranza, Battipaglia, Salerno	0000-0002-9379-4334
Valentina	Sbacco	Università Sapienza di Roma, Dipartimento di Scienze Medico Chirurgiche e Medicina Traslazionale	
Luca	Scaravilli	Ospedale Fornaroli Magenta- ASST Ovest Milanese	00 00000213261862
Andrea	Scardino	IRCCS Istituto Nazionale dei Tumori di Milano - Unità Chirurgia Colorettale	0000-0001-7402-9197
Antonio	Scarpato	-Federico II	
Michela	Schettino	Chirec Hospital Braine-l'Alleud-Waterloo	
Vincenzo	Schiavone	Dipartimento di scienze biomediche avanzate, UOC di Chirurgia Generale ad indirizzo Bariatrico, Endocrino-Metabolico e Senologia,Università degli studi di Napoli Federico II	0000-0002-0533-5833
Elisa	Schirra	Fondazione Policlinico Universitario A. Gemelli IRCCS	
Carlo Maria	Scornajenghi	La sapienza	0000-0001-7600-7434
Virginia	Scorsone	Campus Bio-Medico di Roma	
Marianna	Scribano	Azienda Ospedaliera Policlinico San Marco	
Daniele	Scuto	dipartimento di Chirurgia, Università La Sapienza di Roma	
Kiara	Sejfullai	Fondazione Policlinico Campus BioMedico	
Giuseppe	Sena	A.O.U. "Renato Dulbecco" di Catanzaro	0000-0001-9793-3250
Manfredi Bruno	Sequi	Sapienza Università di Roma, Facoltà di Farmacia e medicina, Polo Pontino	0000-0002-0578-5738
Lorenzo	Serafini	Asienda Ospedaliera IRCCS Sant'Orsola - Malpighi, Bologna	
Giuseppe	Severini	Pineta Grande Hospital	
Enrico	Sicignano	Dipartimento di Neuroscienze e Scienze Riproduttive ed Odontostomatologiche, U.O. Urologia, Università degli Studi di Napoli Federico II	0000-0003-2725-8530
Olga	Silvestri	UOC Chirurgia Vascolare - AOU Federico II Napoli	0009-0005-0195-9023
Leandro	Siragusa	Division of Colon and Rectal Surgery, IRCCS Humanitas Research Hospital, Rozzano, Milano, Italy	0000-0001-9218-1176
Francesco	Smarrazzo	Pineta Grande Hospital	
Federica	Sordelli	IRCCS Humanitas Research Hospital	
Fabiola	Spadafora	UOC Chirurgia plastica ricostruttiva ed estetica Policlinico di Bari	
Maria	Spagnuolo	Policlinico Federico II	
Enrico	Spalice	Department of Surgery, Sapienza University of Rome, Rome 00161, Italy	0009-0009-6220-7588
Marco	Sparavigna	Università degli Studi di Genova	0000-0002-9925-2851
Gaetano Enrico	Spera	Medico in formazione in Ortopedia e Traumatologia	
Alessandra	Spina	Ortopedia e traumatologia Ospedale Sandro Pertini di Roma	
Raffaele	Spinelli	Dipartimento di Neuroscienze e Scienze Riproduttive ed Odontostomatologiche Università degli studi di Napoli Federico II	0000-0001-6551-2080
Lorenzo	Spirito	Vanvitelli	
Chiara	Splendore	/	
Valentina	Stamponi	Asl Umbria 1	0009-0003-4999-8772
Giorgio	Stanziola	AORN ANTONIO CARDARELLI NAPOLI	
Marco	Stizzo	AOU Vanvitelli	
Riccardo	Tajè	Thoracic surgery unit, National Cancer Institute Regina Elena, Rone, Italy	-

Francesco	Taliente	Hepatobiliary Surgery Unit, Fondazione Policlinico Universitario Agostino Gemelli IRCCS, Università Cattolica del Sacro Cuore, Rome, Italy	0000-0003-2681-3889
Simone	Tammaro	UOC UROLOGIA UNIVERSITA DEGLI STUDI DELLA CAMPANIA LUIGI VANVITELLI	
Vincenzo	Tarantino		0009000145090287
Giorgia	Tema	Azienda ospedaliero universitaria Sant andrea	
Claudia	Tempesta	Fondazione Policlinico Campus Biomedico di Roma	
Marco	Terribile	A.O.U. Luigi Vanvitelli	
Matteo	Terrinoni	Dipartimento di Ostetricia e Ginecologia, Università degli Studi di Perugia; Dipartimento di Ostetricia e Ginecologia, USL umbria 1	0009-0009-5087-0374
Ilaria	Tersigni	UOC Chirurgia Generale Oncologica e d'Urgenza Ospedale Belcolle-Santa Rosa Viterbo	0009-0003-2617-9627
Martina	Testicciolі	Fondazione Policlinico Universitario Agostino Gemelli IRCCS-Università cattolica del sacro Cuore	
Marco	Ticonosco	Azienda Ospedaliera Universitaria di Modena	
Ileana	Tiraboschi	Dipartimento di scienze chirurgiche, odontostomatologiche e materno-infantili - UOC di Chirurgia Generele e Epatobiliare - Azienda Ospedaliera Universitaria Integrata Verona	0009-0009-9446-4693
Matteo	Tiracorrendo	Azienda Ospedaliera Sant'Andrea	
Flavio	Tirelli	Fondazione Policlinico Universitario A. Gemelli IRCCS	0000-0001-6160-5992
Mattia	Todaro	Maxillo Facial Surgery Unit, Fondazione Policlinico Agostino Gemelli IRCCS Hospital, Università Cattolica del Sacro Cuore, 8 Largo Agostino Gemelli, 00168 Rome, Italy.	0000-0002-6315-3667
Giovanni	Tomasicchio	Azienda Ospeliero-Universitaria consorziale Policlinico di Bari, Chirurgia Generale Ospedaliera	0000-0001-7017-2690
Giovanni	Tortorelli	IRCCS Istituto Nazionale Tumori Regina Elena	0009-0001-5664-3553
Endri	Toska	Azienda Ospedaliera Universitaria Integrata di Verona	
Valentina	Tosto	Giannina Gaslini Children's Hospital, Department of Obstetrics and Gynecology, Genova, Italy	
Andrea	Totaro	Mako	
Francesco Angelo	Toti	ASST Santi Paolo Carlo	0000-0003-1617-5596
Giorgio	Traina	Università Vita-Salute San Raffaele	0009-0005-6876-6533
Francesco	Trama	Asl napoli 2nord po pozzuoli santa maria delle grazie	
Chiara	Tranfaglia	Specializzanda	
Giovanni	Traumueller Tamagnini	Università Roma La Sapienza	0009-0003-4300-5899
Giuseppe	Trefiletti	Dipartimento Chirmed, UOC di Urologia, Università degli studi di Catania, Azienda Ospedalierouniversitaria Gaspare Rodolico	0009-0007-6101-646X
Lorenza	Trentavizi	Chirurgia generale ospedale di orvieto uslumbria2	
Massimiliano	Trivellato	Aorn Monaldi- ospedali dei colli Napoli	0009-0001-2744-2103
Arianna	Trizzino	UO Chirurgia Epatica e del Trapianto di Fegato - AOU Pisana	0000-0002-6075-175X
Ausilia	Trovato	Humanitas istituto clinico catanese	
Irene	Tucceri Cimini	Department of Biotechnological and Applied Clinical Sciences, General Surgery Unit, San Salvatore Hospital, University of L'Aquila	0000-0001-7763-3357
Piergiorgio	Turco	Azienda ospedaliera universitaria Federico II di Napoli	0000-0002-8073-1537
Giulia	Turri	UOC Chirurgia Generale ed Epatobiliare, Azienda Ospedaliera Universitaria Verona	0000-0001-6150-6812
Filippo	Turri	Clinica Urologica Policlinico Agostino Gemelli	
Irene	Urciuoli	Università di Roma La Sapienza	
Sofia	Usai	Azienda Ospedaliera San Camillo Forlanini	
Matteo	Valente	Azienda ospedaliero-universitaria Sant'Andrea	
Damiano	Vallati	Policlinico Umberto I	
Giampaolo	Valle	Sapienza	
Camilla	Vanni	Thoracic Surgery Division, Sant'Andrea Hospital, Sapienza University of Rome, Roma, Italy	0000-0001-5218-6757
Gianluca	Vanni	dipartimento di scienze chirurgiche- univerisità degli studi di roma tor vergata	0000-0002-3006-5855
Jacopo	Vannucci	University of Perugia Medical School	
Filippo	Varlese	Policlinico Federico 2	
Domenico	Varriale	Federico II Napoli	domvarriale@orcid
Raffaella	Vega	Università di Pisa	
Roberta	Vella	Reparto di chirurgia Epatobiliopancreatica, ospedale Pederzoli, peschiera del Garda, Italia	
Ivan	Vella	Department for the Treatment and Study of Abdominal Diseases and Abdominal Transplantation, Istituto di Ricovero e Cura a Carattere Scientifico-Istituto Mediterraneo per i Trapianti e Terapie ad alta specializzazione (IRCCS-ISMETT), University of Pittsburgh Medical Center (UPMC), Palermo, Italy.	0000-0002-4946-512X

Chiara	Veneroso	Università La Sapienza di Roma	
Luca	Ventrone	Dipartimento di Chirurgia - Università "La Sapienza" di Roma - AOU Policlinico Umberto I di Roma	0000-0003-0131-9120
Gianluca	Villano	Urologia Federico II	
Paolo	Vincenzi	Clinica di Chirurgia epatobiliopancreatica e dei Trapianti, Dipartimento di Gastroenterologia e dei Trapianti, Azienda Ospedaliera Universitaria delle Marche, Ancona	0000-0002-5446-2556
Danilo	Vinci	Hepato-Biliary and Pancreatic Surgery Unit, Department of Surgery, Hospital del Mar, Pompeu Fabra University, Barcelona, Spain	0000-0002-8215-5756
francesco	virgilio	Ospedale San Raffaele	0009-0007-3247-0639
Pietro	Viscuso	San Carlo di Nancy, Roma	0000-0002-1252-1511
Arianna	Vittori	Dipartimento di Scienze Chirurgiche, Oncologiche e Gastroenterologiche, Chirurgia Generale 1, Azienda Ospedale Università Padova	0000-0001-8681-968X
Matteo	Vittori	Urologia Robotica e Mini-Invasiva, Policlinico Tor Vergata Roma	0000-0002-5597-2742
Giuseppe	Vizzielli	Dipartimento di Medicina (DMED), Università degli studi di Udine	0000-0002-2424-2691
Petra	Vogrig	Uosd urologia Policlinico Tor Vergata	0009-0002-9401-9062
Maria Laura	Vuolo	Chirurgia Generale Bariatrica e Metabolica ospedale Santa Maria Nuova, usl toscana centro	0009-0005-5578-8143
Biagio	Zampogna	1) BIOMORF Department, University of Messina, Italy.<br>2) Department of Medicine, Università Campus Bio-Medico di Roma.<br>3) Operative Research Unit of Orthopaedics and Trauma, Fondazione Policlinico Universitario Campus Bio-Medico di Roma.	0000-0002-9318-4114
Alice	Zampolini Faustini	Fondazione Policlinico Universitario Agostino Gemelli	0009-0005-7004-2462
Serena	Zanardo	Dipartimento di scienze cardio-toraco-vascolari e sanità pubblica, Chirurgia Toracica, Azienda Ospedaliera Universitaria di Padova	0009-0003-1020-6862
Nadia	Ziani	Università di Torino, Ospedale San Luigi Gonzaga, Dipartimento di Urologia	0009-0006-2792-3982
Francesco Pio	Zippo	Chirurgia Generale "V. Bonomo" Policlinico di Bari	0009-0001-6224-2852
Andrea	Zironda	ASST Grande Ospedale Metropolitano Niguarda	0000000208741861
Nicolas	Zucchini	Department of Medical, Surgical and Health Sciences, University of Trieste, Strada Di Fiume, 447, 34149, Trieste, Italy	0000000226364094
Davide	Zulian	Chirurgia generale e digestiva, dipartimento di chirurgia generale, Humanitas research hospital	0000-0002-8381-5832
Sara	Negrello	Department of Medical and Surgical Sciences for Children & Adults, Cranio-Maxillo-Facial Surgery, University of Modena and Reggio Emilia, Largo del Pozzo, 71, 41124, Modena, Italy	
Mattia	Manitto	IRCCS Ospedale Policlinico San Martino, 16132 Genova, Italy	
Alessandro	Coppola	Department of Surgery, Sapienza University of Rome, 00161 Rome, Italy	
Giovanni	Comacchio	Azienda Ospedale - Università Padova	
Maria Rosaria	Mastrangeli	Department of Surgery, Sant'Eugenio Hospital, Piazzale Dell'Umanesimo 10, Rome, 00144, Italy.	
Gaetano	Gallo	Colorectal Surgery Unit, IRCCS San Raffaele Scientific Institute, Vita-Salute University, Milan, Italy.	
Antonia	Girardi	Chirurgia Epatobioliopancreatica mininvasiva e robotica ospedale Regionale Miulli Acquaviva delle Fonti	
Edoardo	Pasqui	University of Siena, Vascular Surgery Unit, Siena, Italy	
Eleonora	Guaitoli	Uoc chirurgia generale, PO Valle d'Itria Martina Franca (Ta)	
Rossella	Elia	U.O.C. Chirurgia Plastica, Ricostruttiva ed Estetica, Dipartimento di Medicina di Precisione e Rigenerativa e Area Jonica, Università degli Studi di Bari Aldo Moro, A.O.C. Policlinico di Bari, Bari, Italy	
Luigi	Losco	Dipartimento di Medicina, Chirurgia e Odontoiatria. Università degli studi di Salerno	
Domenico	Soriero	Chirurgia Oncologica Ospedaliera, IRCCS Ospedale Policlinico San Martino, 16132, Genoa, Italy.	
