# Supplementary material for: Robotic surgery across surgical specialties: an Italian survey on implementation, training, and satisfaction levels among trainees and young attendings: on behalf of the Italian Multispecialty Society of Young Surgeons (SPIGC)
Source: Updates Surg. 2026 Sep 7;78(7):2815–35. doi: 10.1007/s13304-026-02747-y (PMC13751746; doi:10.1007/s13304-026-02747-y)
Supplement: Supplementary file 2 [file 13304_2026_2747_MOESM1_ESM.docx]

**Supplementary table 1.** Survey structure and questions

**MODULE 1 - Demographics and General Information**

Q1: Personal details (full name, email, affiliation, ORCID)

Q2: Surgical specialization

Q3: Gender

Q4: Age

Q5: Italian city of practice

Q6: Italian region of practice

Q7: Type of hospital/institution

Q8: Current professional role

Q9: Robot-assisted surgery possibility in specialization

Q10: Interest level in robotic surgery (1-5 scale)

Q11: Co-authorship in SPIGC Collaborative Group

**MODULE 2 - Robotic Surgery Distribution and Utilization**

Q12: Years of robotic platform availability at center?

Q13: Annual robotic surgery procedures performed at center?

Q14: Number of robotic platforms available at center?

Q15: Types of robotic platforms available at center?

Q16: Number of expert surgeons practicing robotic surgery?

Q17: Adequacy of mentoring provided by expert surgeons?

Q18: Dual console availability in operating room?

Q19: Availability of simulation and training software?

Q20: Availability of lectures on robotic platforms and perioperative care?

Q21: Would you recommend your center for robotic surgery training?

Q22: Would you change institution for better robotic surgery training?

Q23: Overall satisfaction with robotic surgery training pathway?

**MODULE 3 - Clinical Involvement in Robotic Surgery**

Q24: Who operates as primary console surgeon during procedures?

Q25: Percentage of trainees operating as primary surgeons vs experts?

Q26: Who serves as bedside assistant during procedures?

Q27: What is your main role during robotic procedures?

Q28: Should robotic surgery be exclusive to expert surgeons?

Q29: Is this affecting your training pathway?

Q30: Monthly procedures performed as console operator?

Q31: Monthly procedures performed as bedside assistant?

Q32: Satisfaction with involvement in robotic procedures?

Q33: Optimal year for trainee involvement in robotic surgery?

Q34: Best methods for robotic surgery training during residency?

Q35: How should robotic surgery competency be assessed?

**MODULE 4 - Training Experiences at Qualified Centers**

Q36: Experience at Italian robotic surgery reference centers?

Q37: Experience at international robotic surgery reference centers?

Q38: Fellowship or post-specialization training availability at your center?

Q39: Should pharmaceutical companies play active role in training?

Q40: Interest in training at other Italian centers?

Q41: Interest in training abroad
